# Multi-centre benchmarking of deep learning models for COVID-19 detection in chest x-rays

**DOI:** 10.3389/fradi.2024.1386906

**Published:** 2024-05-21

**Authors:** Rachael Harkness, Alejandro F. Frangi, Kieran Zucker, Nishant Ravikumar

**Affiliations:** ^1^ School of Computing, University of Leeds, Leeds, United Kingdom; ^2^ Centre for Computational Imaging and Simulation Technologies in Biomedicine, Leeds, United Kingdom; ^3^ Division of Informatics, Imaging, and Data Sciences, School of Health Sciences, University of Manchester, Manchester, United Kingdom; ^4^ Department of Computer Science, School of Engineering, University of Manchester, Manchester, United Kingdom; ^5^ Leeds Institute of Medical Research, School of Medicine, University of Leeds, Leeds, United Kingdom

**Keywords:** deep learning, COVID-19, chest x-rays, artificial intelligence, benchmarking

## Abstract

**Introduction:**

This study is a retrospective evaluation of the performance of deep learning models that were developed for the detection of COVID-19 from chest x-rays, undertaken with the goal of assessing the suitability of such systems as clinical decision support tools.

**Methods:**

Models were trained on the National COVID-19 Chest Imaging Database (NCCID), a UK-wide multi-centre dataset from 26 different NHS hospitals and evaluated on independent multi-national clinical datasets. The evaluation considers clinical and technical contributors to model error and potential model bias. Model predictions are examined for spurious feature correlations using techniques for explainable prediction.

**Results:**

Models performed adequately on NHS populations, with performance comparable to radiologists, but generalised poorly to international populations. Models performed better in males than females, and performance varied across age groups. Alarmingly, models routinely failed when applied to complex clinical cases with confounding pathologies and when applied to radiologist defined “mild” cases.

**Discussion:**

This comprehensive benchmarking study examines the pitfalls in current practices that have led to impractical model development. Key findings highlight the need for clinician involvement at all stages of model development, from data curation and label definition, to model evaluation, to ensure that all clinical factors and disease features are appropriately considered during model design. This is imperative to ensure automated approaches developed for disease detection are fit-for-purpose in a clinical setting.

## 1 Introduction

The unprecedented clinical need arising from the recent severe acute respiratory syndrome coronavirus 2 (SARS-CoV-2) or COVID-19 pandemic prompted considerable interest from within the artificial intelligence (AI) community. Supported by shared data repositories, publicly available anonymised datasets, and open-source software, researchers developed deep learning (DL) systems with the aim of assisting with COVID-19-related clinical tasks. A large volume of research was published for the detection of COVID-19 in chest radiographs, with researchers developing deep learning solutions to assist with the triaging of patients to prioritise primary diagnostic resources i.e., polymerase chain reaction with reverse transcription (RT-PCR) assays or more sensitive imaging techniques e.g., CT scans. With further research investigating the potential for automated imaging-based COVID-19 detection systems as a “second-check” option for cases with suspected false negative RT-PCR results, a consequence of this test’s well-documented low sensitivity (1, 2). RT-PCR is also reported to struggle with false positives in recovered patients during long-term follow-up (3). The application of CXRs can overcome this problem, facilitating differentiation between ongoing infection and resolving infection.

However, upon a closer review of the literature, we observed serious limitations associated with this area of research. This has brought into question the credibility of model performance claims and prompted several critical reviews of the use of artificial intelligence in this area (4, 5). These reviews identified the models they considered as being at significant risk of bias and attribute this to the pervasive use of poor-quality open-source datasets (5), in combination with insufficient model evaluation (4). A common limitation of existing studies is a lack of sufficient reporting regarding data provenance and quality, and insufficient external validation of developed DL algorithms i.e., most approaches are validated using data from the same centre. Whilst a wide variety of approaches have been proposed for the purpose of identifying risk of bias in models trained on open-source data, there is an absence of studies that perform rigorous evaluative comparisons of models trained on multi-centre hospital data. To address these shortcomings we undertake a comprehensive benchmarking study that evaluates the performance of recently published DL models (4).

We validate these models, under clinical guidance, by considering the practical challenges of interpreting chest x-rays in suspected COVID-19 cases. Perhaps foremost of these challenges is that COVID-19 infection often does not always develop into COVID-19 pneumonia, in which case diagnostic features of COVID-19 cannot be observed in the CXR. Moreover, where COVID-19 pneumonia can be observed, its heterogeneous presentation mimics a broad spectrum of lung pathologies, making it difficult to identify COVID-19 pneumonia due to confounding conditions. The presence of co-occurring conditions, or comorbidities, can also complicate the detection of COVID-19, especially in cases where the disease is mild and features are subtle. Furthermore, the unpredictable temporal progression of COVID-19 presents a challenge for radiologists, as unexplained rapid advancements in the disease and low resolution in chest x-rays (CXRs) contribute to ambiguity (6). Collectively, these factors lead to substantial diagnostic uncertainty when using medical imaging for the detection of COVID-19. We conduct a thorough evaluation of model error scrutinising these factors to better understand model failures, the demographics of those affected, and potential avenues for improvement.

This article presents a comprehensive benchmarking study comparing state-of-the-art DL methods and conducting exhaustive model evaluations on independent, multi-national clinical datasets, with the goal of identifying model strengths and weaknesses while assessing the suitability of automated DL systems as clinical decision support tools in COVID-19 detection.

## 2 Materials and methods

In this section, we present our methodology, providing detailed descriptions of the evaluated models, our training procedures, our evaluation methods, and a thorough review of the datasets used.

### 2.1 Overview of the experimental approach

We utilise two independent UK-based datasets and a further dataset from outside the UK. We train a diverse set of deep learning models on one of the UK-based datasets (NCCID) and validate national generalisability using data from the other UK-based dataset, which is from an independent hospital site, the Leeds Teaching Hospital Trust (LTHT). We consider international generalisability using open-source data from a Spanish hospital (COVIDGR).

We investigate model performance variation by patient-level factors e.g., demographic and smoking history. We also evaluate model vulnerability to confounding variables, which requires the use of counterfactual datasets created from a subset of the LTHT population for whom non-COVID-19 pneumonia status was recorded. In this population we modified the definition of the positive and control classes, resulting in two additional counterfactual datasets. The first dataset referred to as *LTHT PNEUMONIA (P)* simulates a pneumonia detection setting where the positive class includes non-COVID-19 pneumonia cases i.e., no distinction is drawn between COVID-19 and other pneumonia types. The second scenario named *LTHT NO PNEUMONIA (NP)* replicates a COVID-19 detection scenario where all instances of non-COVID-19 pneumonia were deliberately excluded.

Following primary evaluation, we identify the top-performing models for further analysis. We train and validate the best models on region-of-interest (ROI)-extracted CXRs to test whether overall performance of COVID-19 detection is improved with the use of ROIs and if, as is commonly assumed, cropping to the ROI helps to mitigate any inherent data biases. Furthermore, we apply explainable AI techniques to examine highlighted features, i.e., features significant to model prediction. Identification of certain features can indicate model reliance on spurious correlations, which can lead to poor generalisation. The presence of these “shortcut” features has been identified in prior work on models trained with open-source data, we evaluate NCCID-trained models for reliance on similar “shortcut” features (5).

### 2.2 Data

**Figure 1:**
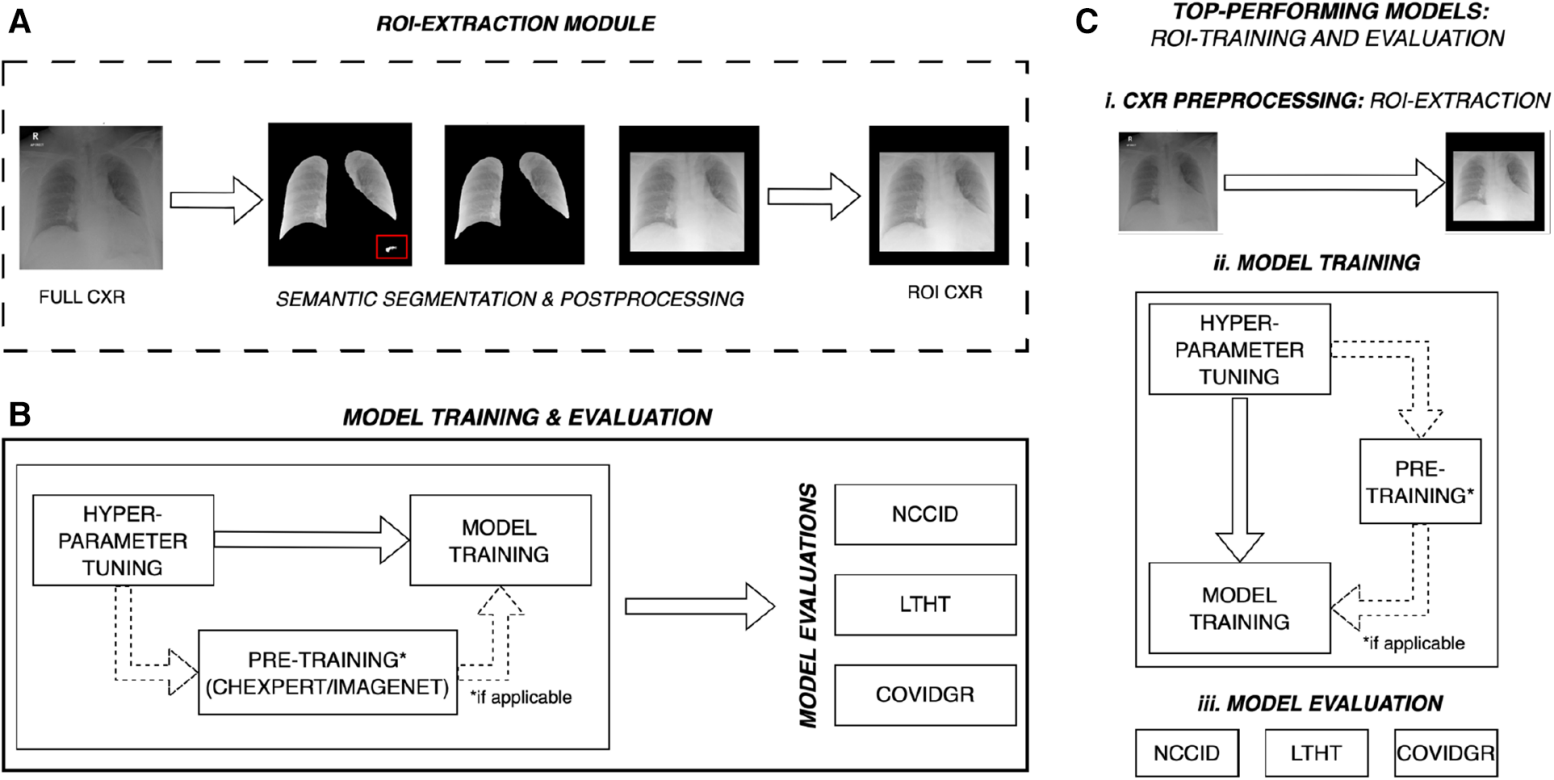
Overall experimental design for multi-centre evaluation of COVID-19 detection models. (**A**) ROI-cropped CXRs are generated from semantic segmentations of the left and right lung fields, automated prediction uncertainty-based post-processing is applied to ensure reliable cropping for both classes of CXR. The red box highlights over-segmentation of the lung fields, post-processing removes this structure prior to extracting the region of interest. (**B**) Some models are pre-trained, for these models hyper-parameters are tuned on ImageNet or CheXpert data (domain-specific dataset). After pre-training, model hyper-parameters are refined for the COVID-19 detection task and trained on full CXRs from the NCCID. Models are subsequently evaluated on three independent populations: the unseen NCCID population, the LTHT, and COVIDGR. (**C**) Following primary training and evaluation, the best performing models are selected for (ii) training and (iii) evaluation on ROI-extracted CXRs. NCCID, National COVID-19 Chest Imaging Database; LTHT, Leeds Teaching Hospital Trust; ROI, Region of Interest; CXR, Chest x-ray.

This study utilises three independent datasets, NCCID, COVIDGR sourced from a hospital in Spain, and a purpose built single site dataset derived from Leeds Teaching Hospitals NHS Trust (LTHT). The NCCID dataset is available upon request, COVIDGR can be found online and the LTHT dataset is not available publicly, however the hospital has a formal data access process through which researchers may apply (Figure 1).

**Figure 2:**
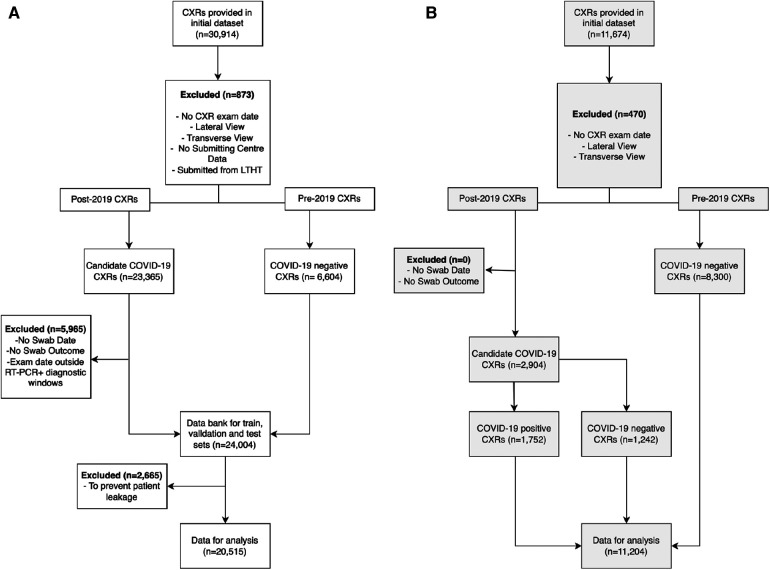
Exclusion criteria for pre-processing (**A**) NCCID and (**B**) LTHT datasets. CXRs were excluded if missing crucial acquisition data (exam date and submission centre) and if not frontal view (AP or PA). NCCID CXRs were eliminated if submitted from Leeds-based hospitals. CXRs were divided into two cohorts: pre-2019 and post-2019. Pre-2019 CXRs were automatically labelled COVID-19 negative, while post-2019 CXRs were evaluated for COVID-19 outcomes. Post-2019 CXRs were eliminated if missing data essential for determining COVID-19 outcome i.e., CXR acquisition date, RT-PCR swab date or outcome. CXRs were also excluded if exam date fell between diagnostic windows of multiple positive RT-PCR swabs. The COVIDGR dataset is not subject to the same exclusion criteria due to a lack of patient data. As a pre-prepared dataset, some exclusion criteria is already applied i.e., COVIDGR includes only PA CXRs. NCCID, National COVID-19 Chest Imaging Database; LTHT, Leeds Teaching Hospital Trust; AP, Antero-posterior; PA, Postero-anterior; CXR, Chest x-ray.

Uniform exclusion criteria were applied to the NCCID and LTHT datasets. CXRs were excluded if case data was insufficient to confidently assign a COVID-19 label, e.g., missing RT-PCR swab date or missing RT-PCR test result data for CXRs collected post-2019. Inclusion criteria was considered using data collected from Digital Imaging and Communication in Medicine (DICOM) headers and associated radiology reports. Note that the international dataset (COVIDGR) did not include RT-PCR swab date or CXR exam date data. Instead, CXR labels were pre-defined with CXRs considered positive if acquired 24 h before or after a positive COVID-19 swab. The labelling schema for all datasets are described in the Supplementary Materials and outlined in Supplementary Figure S1. For all datasets, only frontal CXRs, antero-posterior (AP) and postero-anterior (PA), were included and only clinical testing (SARS-CoV-2 RT-PCR) results were used in producing COVID-19 labels, radiological features indicative of COVID-19 infection were not considered. Figure 2 presents a CONSORT diagram describing the full exclusion criteria applied during data preparation for this study.

#### 2.2.1 Pre-training data (ImageNet & CheXpert)

Pre-trained models were trained on either ImageNet natural images or domain-specific CheXpert CXRs prior to NCCID training (as dictated by original model implementations). ImageNet is a large-scale image classification dataset comprising 14 million annotated natural images from more than 21,000 classes e.g., hummingbird, hen, lion etc. ImageNet is publicly accessible and available for download. CheXpert is a large dataset containing 224,316 chest x-rays from 65,240 patients, each image has recorded outcomes for 14 observations, such as, pleural effusion, cardiomegaly and consolidation (generated from radiology reports) (7). CheXpert is also publicly accessible and available for download.

#### 2.2.2 Training data (NCCID)

The National COVID-19 Chest Imaging Database (NCCID) is a centralised UK database derived from 26 hospital centres, storing 45,635 CXRs from 19,700 patients across the UK in the form of DICOM image files and header information (de-identified). To preserve the independence of our single-site evalution dataset (LTHT), we excluded from NCCID all cases originating from the Leeds area, leaving CXRs collected from 25 different hospital centres. The removed CXRs were neither utilised for model training nor model evaluation on NCCID. NCCID CXRs are provided alongside clinical data, including the results of RT-PCR tests. Dates for both CXR exams and RT-PCR swabs are provided. If exam date or RT-PCR dates were unavailable, the CXR was excluded from the study. RT-PCR was used to define ground truth labels for CXRs. As no standard recognised definition exists within the literature, we sought expert opinion from a radiologist, a respiratory physician, and a clinical oncologist to inform our definition of COVID-19 positive CXRs. We treated CXRs with a positive COVID-19 RT-PCR test anywhere from 14 days before to 28 days after image acquisition as COVID-19 positive. We treated images without a positive RT-PCR test within this diagnostic window as COVID-19 negative (Supplementary Figure S1). After data preparation, the NCCID training dataset consists of 20,515 exams, with 8,337 positive exams and 12,178 control CXRs. Figure 2 presents a CONSORT diagram outlining the full exclusion criteria applied to both NCCID and LTHT datasets.

#### 2.2.3 Testing data (LTHT & COVIDGR)

External validation data is collected from two independent sources, LTHT, a UK-based hospital in Leeds (nationally-sourced), and COVIDGR, made up of CXRs from San Cecelio University Hospital in Granada, Spain (internationally-sourced). LTHT provides patient CXR images (DICOMs), with RT-PCR test results for COVID-19 diagnosis. In LTHT, RT-PCR date is provided relative to CXR exam date to allow precise classification of COVID-19 status according to our chosen diagnostic window (Supplementary Figure S1). The exclusion criteria for LTHT and COVIDGR datasets is summarised in Supplementary Table S1).

Additionally, for a subset of LTHT patients, non-COVID-19 pneumonia diagnostic status was available, from this subset of the LTHT population the counterfactual datasets LTHT (P) and LTHT (NP) were created. To create LTHT (NP) all participants with recorded non-COVID-19 pneumonia were removed from the LTHT dataset. To construct the LTHT (P) dataset the image labelling criteria was changed such that all CXRs positive for pneumonia (COVID-19 or non-COVID-19 pneumonia) were labelled positive. We do this to evaluate the models’ capacity to separate COVID-19 from non-COVID-19 pneumonia cases, a major confounding pathology. For both LTHT (P) and LTHT (NP) populations, participants without non-COVID-19 labels were not considered. The generation of counterfactual datasets is summarised in Supplementary Figure S2.

The COVIDGR dataset provides a total of 852 CXRs sourced from the San Cecelio University Hospital in Granada, Spain. The dataset is balanced, containing 426 positive and 426 negative CXRs. In the creation of the COVIDGR dataset CXRs were chosen through manual selection. COVID-19 CXRs are defined by a positive RT-PCR test, conducted within 24 h of the CXR exam. All CXRs were pre-cropped prior to being compiled into COVIDGR. COVIDGR includes only postero-anterior (PA) views which were acquired with the same scanner type. In addition, RALE severity scores are provided for all positive cases, as well as 76 CXRs in which COVID-19 is not observed (NORMAL-PCR+), 100 mild (MILD), 171 moderate (MODERATE) and 79 serious (SEVERE) cases.

### 2.3 Models

The models we selected for this benchmarking study are diverse in design and leverage different learning paradigms i.e., supervised, transfer, semi-supervised and self-supervised learning (Table 1 and Supplementary Figure S3). We found that the majority of proposed DL methods for COVID-19 detection in CXRs rely on supervised or transfer learning. Here we define supervised models as models trained for COVID-19 detection from randomly initialised weights. All transfer learning approaches used weights pre-trained on either ImageNet or CheXpert and were later fine-tuned in a fully supervised manner on the training dataset (NCCID) for the task of COVID-19 detection. Further details on model selection criteria and model training procedures can be found in the Supplementary Materials.

**Table 1:** Summary of the evaluated models.

Model	References	Abbrvs.	DL type	Pre-trained [Y/N]	Params.
				***(Data)***	(#)
Deep CNN generated by NAS	(10)	COVIDNET	Supervised	Y (CheXpert)	50,150,485
Multiscale attention guided network with soft distance regularisation	(12)	MAG-SD	Supervised	Y (ImageNet)	23,835,968
Vision transformer	(17)	XVITCOS	Supervised	Y (CheXpert)	86,537,477
Ensemble of deep CNNs	(13)	FUSENET	Supervised	Y (ImageNet)	17,245,921
Deep CNN with Xception backbone	(8)	XCEPTION NET	Supervised	Y (ImageNet)	21,331,753
Deep CNN with residual connections and attention component	(11)	RES. ATTN.	Supervised	N	5,476,673
Deep CNN with EfficientNet backbone	(9)	ECOVNET	Supervised	Y (ImageNet)	7,304,737
Convolutional capsule network	(14)	CAPSNET	Supervised	Y (CheXpert)	523,072
Convolutional autoencoder with classifier	(15)	CORONET	Semi-supervised	Y (ImageNet)	11,230,978
Deep CNN with attention mechanism, pre-trained under self-supervised conditions	(16)	SSL-AM	Self-supervised	Y (CheXpert)	6,753,905

Models are described and presented alongside source reference, pre-training status and deep learning category. Models are referred to by their designated abbreviations. DL, Deep Learning; NAS, Neural Architecture Search; Params., Parameters.

Within the supervised learning category, we explored the use of various well-established deep convolutional neural network (CNN) backbones. Of these, we identified XCEPTION NET (8) and ECOVNET (9) from highly cited publications as influential models of interest. Similar approaches place emphasis on domain-specific tuning, rather than applying a pre-defined deep CNN backbone. For example, COVIDNET (10) is defined by a generative neural architecture search (NAS) for optimal COVID-19 detection performance. Other deep CNN approaches employ unique designs to encourage the recognition of domain-specific features, such as RES. ATTN. (11) which incorporates attention mechanisms, and MAG-SD (12) which uses hierarchical feature learning. Although all of these approaches share some similarities training strategies differ. For each model, we reproduce the pre-training strategies outlined in their respective studies in order to maintain consistency with first reported implementations. Under the supervised learning category, we also include a vision transformer (XVITCOS), a deep CNN-ensemble network (FUSENET) (13), and a capsule network (CAPSNET) (14).

We select CORONET (15) as an example of semi-supervised learning. CORONET relies on a two stage process to classify images, comprising a convolutional autoencoder in the first stage and a standard CNN classifier in the second stage (Supplementary Figure S3). First, the convolutional autoencoder is trained to reconstruct COVID-19 negative CXRs from learned low-dimensional latent representations. The classifier is then trained to predict CXR outcomes taking images comprising the pixel-wise differences between original CXRs and autoencoder reconstructions (residual images) as inputs. The intuition is that the reconstructions of CXRs from the unseen class (COVID-19 positive) will fail to preserve radiographic features of COVID-19 infection, which will appear in residual images. Other approaches like SSL-AM (16), follow a self-supervised pre-training strategy. In SSL-AM, representations learned during pre-training are enhanced through 2D transformations, such as, distortion, in-painting and perspective transformations. During pre-training, SSL-AM is comprised of a UNet-style network architecture which learns domain-specific features independent of the disease class. Following pre-training, the decoder portion of the UNet is subsequently discarded, while, the encoder and its pre-trained, domain-specific weights are incorporated into a COVID-19 classifier.

### 2.4 Model training

We apply a pre-defined training protocol designed to facilitate uniform comparison in model performance. We train models on NCCID training data across 5-fold cross-validation experiments, each of which comprises a balanced number of COVID-19 negative and COVID-19 positive cases. Prior to training we, where necessary, adapt the original models for our task of binary classification of CXRs, i.e., to accommodate larger image resolution (than was used in the original implementation of any of the selected models) or to predict two classes instead of three.

We used the CheXpert dataset for models that required pre-training on domain-specific datasets. Specifically, model weights were optimised for the task of predicting lung pathologies in CXRs.

Models that required pre-training on natural images were pre-trained on ImageNet. The choice of dataset, and if pre-training is even required, is dictated by the original model implementation. For all training stages, images were resized to 480x480 and standard image transformations were applied. We also tune the learning rates for each model, at each stage of training, using Optuna which is an open source hyperparameter optimisation framework (for further details see Supplementary Materials). As models are identified from pre-existing, published works we accept model architecture hyper-parameters as fixed and do not tune these to the training datasets.

#### 2.4.1 Lung segmentation (ROI)

**Figure 3:**
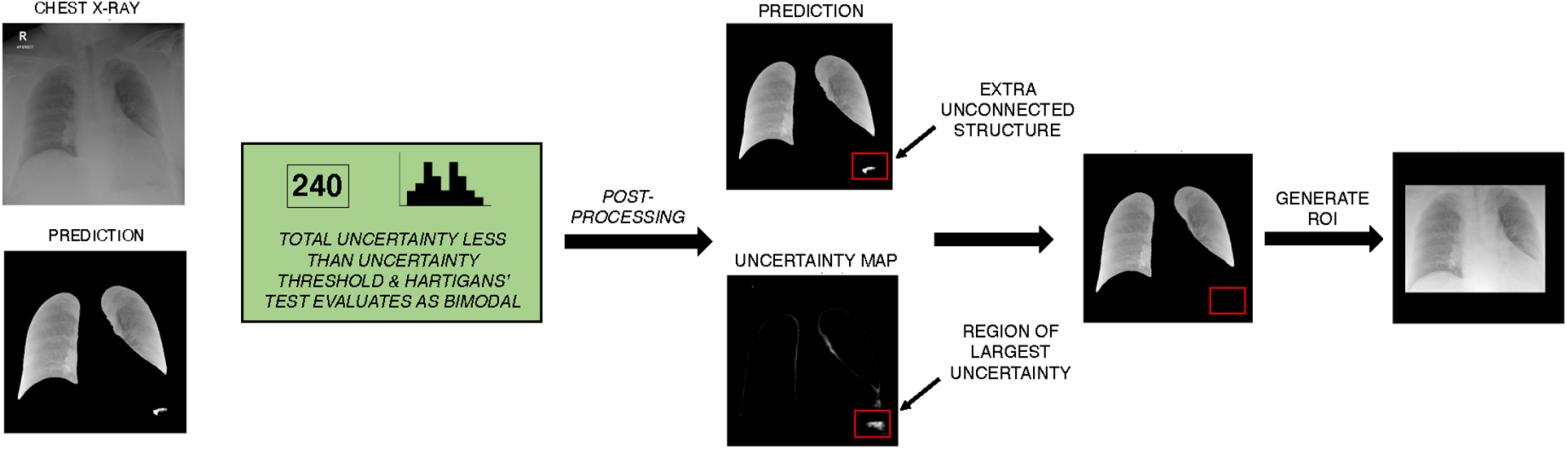
Example of unsupervised lung segmentation post-processing algorithm on NCCID data. The UNet++ model is used to generate semantic segmentation of the left and right lung field. Monte Carlo dropout is applied to approximate uncertainty of prediction, total uncertainty is calculated and frequency of uncertainty is evaluated for Bimodality with Hartigans’ test. In this example, uncertainty is less than the threshold for required manual inspection and prediction uncertainty is bimodal so automatic post-processing is applied. In post-processing, unconnected structures are identified and the density of uncertainty is calculated per structure. Excess structures (more than the two lung fields) are iteratively removed, with the most uncertain structures removed first. A ROI is generated from the post-processed semantic segmentation, the ROI was selected to be the minimum bounding box around the segmented lung fields. ROI, Region of interest; NCCID, National COVID-19 Chest Imaging Database.

Automatic segmentation of lung fields is often applied to mitigate the influence of confounding variables and background artefacts/noise. To test this, the top three performing models are also trained using CXRs cropped to the lung fields, which have been separated from background tissue using semantic segmentation. To generate these segmentations we trained a UNet++ model on the open-source dataset COVID-QU-Ex, containing 33,920 COVID-19, pneumonia, and normal CXRs, all with ground truth segmentation masks (further details provided in the Supplementary Material) (18). To improve segmentation robustness and reduce the risk of introducing a segmentation quality bias to the downstream classification task, we applied a novel epistemic uncertainty-based post-processing algorithm to revise predictions or flag predictions for manual inspection where necessary. For the task of lung field segmentation, correct segmentations are expected to comprise two connected components, each component corresponding to the left or right lung field. Additionally, successful segmentations are assumed to have corresponding pixel-wise prediction uncertainty maps that are unimodal with uncertainty predominantly concentrated along the borders of the lungs. This is what we would expect to observe if a panel of radiologists were tasked with outlining lung fields in CXRs (and inter-rater variability/uncertainty was quantified). Thus, we also assume that a bimodal uncertainty frequency is evidence of erroneous segmentation outside normal inter-rater variability. If predicted segmentation masks are found to have more than two unconnected components, their corresponding uncertainty maps were then assessed for Bimodality using Hartigans’ dip test. Predictions that produce bimodal pixel-wise uncertainty frequency distributions, and give a total uncertainty below an empirically defined threshold, are highlighted as likely erroneous predictions and excess structures are iteratively eliminated according to greatest total uncertainty per segmented area i.e., structures with the greatest density of uncertainty are removed first. Predictions that exceed the total uncertainty limit are put forward for manual inspection. As a result of preliminary experiments, we applied a total uncertainty limit of 800, which we found facilitated selection of the best candidates for post-processing. Once this process is applied, we crop CXRs to the remaining segmented areas, this produces our region of interest (ROI). We use ROI instead of semantic segmentation for added robustness and to ensure that all clinically significant thoracic structures are included e.g., the mediastinum (Figure 3).

With a total uncertainty threshold of 800, region of interest (ROI) prediction Dice scores improved from 0.96 to 0.98. While improvements in scores on data from the same training distribution are modest, it is expected that applying the proposed uncertainty-based post-processing algorithm will help improve overall ROI-extraction accuracy for CXR data from unseen domains. Qualitative evaluation of segmentations performed on the NCCID training data showed that applying the post-processing algorithm improved the accuracy and robustness of predicted ROIs (Supplementary Figure S4).

### 2.5 Performance evaluation

We evaluated predictive performance on multiple independent test populations, with classification thresholds set to 0.5 for ease of comparison. To compare the classification performance of all models, we evaluated performance metrics, such as, accuracy, precision, recall, F1, and AUROC. To consider average performance over all iterations of the 5-fold cross-validation, we calculated confidence intervals for all ROC curves and mean ± standard deviations for classification metrics. Models were ranked according to their individual performance metrics and all metric rankings were considered equally to give an overall model ranking. We perform Tukey’s honestly significant difference (HSD) statistical test to compare model performance. We investigated model explanation techniques, including GradCAM and guided backpropagation visualisation methods. Additionally, we trained the top-performing models on ROI-extracted CXRs, allowing us to directly compare these ROI-trained models with their counterparts trained on the entire CXR.

#### 2.5.1 Model evaluation in national and international hospital populations

Model capacity to generalise to national populations was evaluated using external NHS hospital data from LTHT. With this evaluation we estimate how the models perform in an unseen hospital trust, in which patient demographics and clinical practices may vary.

Furthermore, we conducted an assessment of model generalisability to international hospital populations, utilising data from the Grenada Hospital in Spain (referred to as COVIDGR). Note that our evaluation of international generalisability is limited due to an uncontrollable label shift across patient populations (a consequence of different labelling strategies).

##### 2.5.1.1 Model performance under counterfactual conditions

We also created counterfactual datasets from a subset of LTHT data for which we also have non-COVID-19 pneumonia labels. We adjusted the definition of the positive and control classes in LTHT data, resulting in the creation of two alternative scenarios for comparison of model performance under counterfactual conditions. The first scenario, referred to as LTHT (P), encompasses a general pneumonia detection scenario where the positive class includes non-COVID-19 pneumonia CXRs. The second scenario, named LTHT (NP), represents a COVID-19 detection scenario where all instances of non-COVID-19 pneumonia were excluded. We evaluate models according to standard performance metrics and perform sub-population analysis under counterfactual conditions.

##### 2.5.1.2 Model performance variation by patient-level factors

Sub-population analysis was performed on LTHT data. We assessed model performance across different patient sub-populations, grouped according to ethnicity, age, sex, smoking status, and the presence of comorbidities within the CXR. To create CXR-observable comorbidity subgroups we convert patient recorded comorbidities into a binary label that describes whether the comorbidity is likely observable in the CXR. More detail on this is available in Supplementary Materials. Moreover, ethnic subgroups are defined according to NHS ethnic categories, which we in turn group into five larger populations: Black, White, Asian, Multiple and Other. “Other” describes any ethnicity that does not fall under the aforementioned ethnic categories, cases with unknown ethnicity are not considered in our analysis.

We perform one-way analysis of variance (ANOVA) tests to evaluate the statistical significance of differences in model performance across different subgroups. We also assessed model error rate and its correlations with various clinical and demographic factors. We examined the effects of CXR projection, RALE-defined CXR severity, and proximity to the COVID-19 diagnostic window on the rates of false positive and false negative predictions. Refer to Table 2 for a summary of which dataset is used for each specific task.

**Table 2:** Overview of individual dataset use throughout this study, including ROI-extraction, model training and evaluations.

	NCCID (**TRAIN**)	NCCID (**TEST**)	LTHT	COVIDGR	COVID-QU-Ex
Lung segmentation model training & testing					✓
Model training	✓				
Model performance evaluation	✓	✓	✓	✓	
Sub-population analysis			✓		
Counterfactual evaluation			✓(subset w/known pneumonia outcomes)		
RALE-dependent performance evaluation				✓	

For evaluation of models under counterfactual conditions we used a subset of LTHT with recorded non-COVID-19 pneumonia status. NCCID, National COVID-19 Chest Imaging Database; LTHT, Leeds Teaching Hospital Trust; ROI, Region of Interest; RALE, Radiographic Assessment of Lung Edema.

## 3 Results

In this section we present the results of our study, providing a comprehensive description of the key findings and observations drawn from the analysis of the considered models and datasets.

### 3.1 Evaluation of model performance in national and international hospital populations

All models generalised well to a national-level but perform poorly when applied to international datasets. Table 3 shows that there is a marginal decrease in model performance when applied beyond the training domain (NCCID) to the unseen NHS trust population (LTHT).

**Table 3:** Average model performance metrics for each test dataset, with standard deviation, across cross-validation folds.

		Models									
		COVIDNET	MAG-SD	XVITCOS	FUSENET	XCEPTION NET	RES. ATTN.	ECOVNET	COVID-CAPS	CORONET	SSL-AM
**NCCID TEST**	*Acc.*	0.66 ± 0.04	0.63 ± 0.01	0.67 ± 0.03	0.65 ± 0.02	**0.70 ± 0.02**	0.59 ± 0.04	0.67 ± 0.02	0.56 ± 0.03	0.67 ± 0.03	0.69 ± 0.03
	*Prec.*	0.50 ± 0.04	0.46 ± 0.01	0.49 ± 0.03	0.47 ± 0.02	**0.53 ± 0.02**	0.42 ± 0.03	0.50 ± 0.02	0.40 ± 0.01	0.50 ± 0.04	0.52 ± 0.03
	*Recall*	0.63 ± 0.12	**0.78 ± 0.01**	0.73 ± 0.07	0.64 ± 0.07	0.69 ± 0.04	0.61 ± 0.11	0.66 ± 0.03	0.69 ± 0.05	0.64 ± 0.10	0.70 ± 0.05
	*F1*	0.54 ± 0.01	0.58 ± 0.00	0.59 ± 0.01	0.54 ± 0.02	**0.60 ± 0.01**	0.49 ± 0.02	0.57 ± 0.02	0.51 ± 0.01	0.56 ± 0.02	0.59 ± 0.01
	*AUROC*	0.70 ± 0.01	0.73 ± 0.01	0.74 ± 0.01	0.70 ± 0.01	**0.75 ± 0.01**	0.63 ± 0.01	0.71 ± 0.01	0.65 ± 0.01	0.72 ± 0.01	0.74 ± 0.02
N=824	*Ranking*	7	3	6	8	2	10	5	9	4	1
**LTHT**	*Acc.*	0.68 ± 0.04	0.68 ± 0.04	0.70 ± 0.03	0.65 ± 0.08	**0.75 ± 0.01**	0.65 ± 0.04	0.75 ± 0.01	0.66 ± 0.04	0.72 ± 0.04	0.72 ± 0.03
	*Prec.*	0.28 ± 0.03	0.28 ± 0.03	0.30 ± 0.02	0.26 ± 0.03	**0.34 ± 0.01**	0.23 ± 0.02	0.32 ± 0.02	0.24 ± 0.02	0.28 ± 0.04	0.30 ± 0.03
	*Recall*	0.64 ± 0.05	0.63 ± 0.06	**0.66 ± 0.04**	0.62 ± 0.10	0.65 ± 0.02	0.53 ± 0.07	0.52 ± 0.03	0.52 ± 0.05	0.52 ± 0.16	0.62 ± 0.03
	*F1*	0.38 ± 0.01	0.39 ± 0.03	0.41 ± 0.01	0.36 ± 0.02	**0.45 ± 0.01**	0.32 ± 0.02	0.39 ± 0.02	0.32 ± 0.01	0.36 ± 0.06	0.41 ± 0.03
	*AUROC*	0.72 ± 0.01	0.73 ± 0.03	0.75 ± 0.00	0.70 ± 0.01	**0.78 ± 0.01**	0.65 ± 0.03	0.72 ± 0.02	0.65 ± 0.01	0.70 ± 0.05	0.74 ± 0.03
N=11,204	*Ranking*	6	5	2	8	1	10	4	9	7	3
**LTHT (NP)**	*Acc.*	0.71 ± 0.04	0.70 ± 0.05	0.74 ± 0.03	0.68 ± 0.08	**0.75 ± 0.02**	0.59 ± 0.07	0.62 ± 0.04	0.58 ± 0.04	0.60 ± 0.15	0.69 ± 0.03
	*Prec.*	0.96 ± 0.01	0.96 ± 0.01	0.97 ± 0.01	0.96 ± 0.01	**0.98 ± 0.00**	0.95 ± 0.01	0.98 ± 0.01	0.96 ± 0.01	0.97 ± 0.01	0.96 ± 0.01
	*Recall*	0.71 ± 0.05	0.69 ± 0.06	0.73 ± 0.04	0.68 ± 0.10	**0.73 ± 0.02**	0.57 ± 0.07	0.59 ± 0.04	0.56 ± 0.05	0.57 ± 0.17	0.68 ± 0.03
	*F1*	0.81 ± 0.03	0.80 ± 0.04	0.83 ± 0.02	0.79 ± 0.07	**0.84 ± 0.01**	0.71 ± 0.06	0.73 ± 0.03	0.71 ± 0.04	0.70 ± 0.15	0.80 ± 0.03
	*AUROC*	0.80 ± 0.02	0.78 ± 0.05	0.83 ± 0.01	0.76 ± 0.02	**0.88 ± 0.02**	0.73 ± 0.06	0.83 ± 0.02	0.75 ± 0.03	0.80 ± 0.06	0.79 ± 0.05
N=3,948	*Ranking*	3	6	2	8	1	9	4	10	7	5
**LTHT (P)**	*Acc.*	0.54 ± 0.05	0.53 ± 0.06	**0.55 ± 0.04**	0.55 ± 0.10	0.52 ± 0.02	0.47 ± 0.06	0.45 ± 0.02	0.46 ± 0.05	0.43 ± 0.12	0.49 ± 0.02
	*Prec.*	0.98 ± 0.00	0.98 ± 0.01	0.99 ± 0.00	0.98 ± 0.00	**0.99 ± 0.00**	0.98 ± 0.01	0.99 ± 0.00	0.98 ± 0.00	0.99 ± 0.01	0.98 ± 0.00
	*Recall*	0.53 ± 0.06	0.52 ± 0.06	**0.54 ± 0.04**	0.54 ± 0.11	0.50 ± 0.02	0.46 ± 0.07	0.43 ± 0.02	0.45 ± 0.06	0.41 ± 0.13	0.48 ± 0.03
	*F1*	0.69 ± 0.05	0.68 ± 0.05	**0.70 ± 0.04**	0.69 ± 0.09	0.67 ± 0.02	0.62 ± 0.06	0.60 ± 0.02	0.61 ± 0.05	0.57 ± 0.13	0.65 ± 0.02
	*AUROC*	0.69 ± 0.03	0.67 ± 0.04	0.72 ± 0.02	0.67 ± 0.02	**0.75 ± 0.03**	0.66 ± 0.06	0.75 ± 0.02	0.69 ± 0.03	0.70 ± 0.05	0.66 ± 0.04
N=1,451	*Ranking*	3	6	1	4	2	10	5	9	7	8
**COVIDGR**	*Acc.*	0.58 ± 0.01	0.62 ± 0.01	0.61 ± 0.02	0.58 ± 0.02	**0.63 ± 0.01**	0.56 ± 0.04	0.58 ± 0.04	0.55 ± 0.02	0.56 ± 0.03	0.59 ± 0.03
	*Prec.*	0.73 ± 0.06	0.71 ± 0.06	0.73 ± 0.04	0.80 ± 0.08	0.86 ± 0.05	0.59 ± 0.06	**0.90 ± 0.07**	0.62 ± 0.04	0.66 ± 0.07	0.78 ± 0.05
	*Recall*	0.31 ± 0.11	**0.42 ± 0.10**	0.34 ± 0.04	0.23 ± 0.09	0.30 ± 0.02	0.38 ± 0.11	0.18 ± 0.09	0.27 ± 0.07	0.26 ± 0.11	0.26 ± 0.10
	*F1*	0.42 ± 0.08	**0.51 ± 0.06**	0.46 ± 0.04	0.34 ± 0.11	0.44 ± 0.02	0.46 ± 0.10	0.29 ± 0.13	0.37 ± 0.07	0.36 ± 0.12	0.38 ± 0.10
	*AUROC*	0.63 ± 0.03	0.67 ± 0.02	0.67 ± 0.03	0.63 ± 0.01	**0.71 ± 0.02**	0.59 ± 0.10	0.70 ± 0.03	0.60 ± 0.03	0.60 ± 0.05	0.67 ± 0.01
N=852	*Ranking*	6	2	3	7	1	8	5	9	10	4

N is the total number of cases in each test population. **Bold** indicates the highest average metric per dataset. NCCID, National COVID-19 Chest Imaging Database; LTHT, Leeds Teaching Hospital Trust; Acc, Accuracy; Prec, Precision; AUROC, Area Under the Receiver Operator Characteristic.

During LTHT population testing AUROC scores ranged from 0.65 to 0.78. XCEPTION NET (8), XVITCOS (17) and SSL-AM (16) emerged as top-performing models, with AUROCs between 0.74 and 0.78. We identify RES. ATTN. (11), CAPSNET (14), and FUSENET (13) as the poorest performing models. Table 3 shows that RES. ATTN., the only model without a pre-training strategy, gives the lowest performance across all evaluated metrics. RES. ATTN., FUSENET and CAPSNET AUROCs scores are lower than all other models, this is statistically significant to a confidence interval of 95% (Tukey multiple comparisons tests, p<0.05; Supplementary Table S4).

Even top-performing models are susceptible to returning high rates of false positives, as evidenced by universally low precision scores (Table 3). However, even without classification threshold tuning, the top-performing models detect COVID-19 in LTHT populations similarly to radiologist performance in a variety of performance metrics. Model accuracy scores ranged from 0.69 to 0.75 and one study reports the average accuracy scores of radiologist groups as between 0.76 to 0.84, depending on professional experience (19). Comparison with another study shows that the best performing model AUROCs exceed radiologist performance, with scores of 0.78 compared to 0.71 (20).

**Figure 4:**
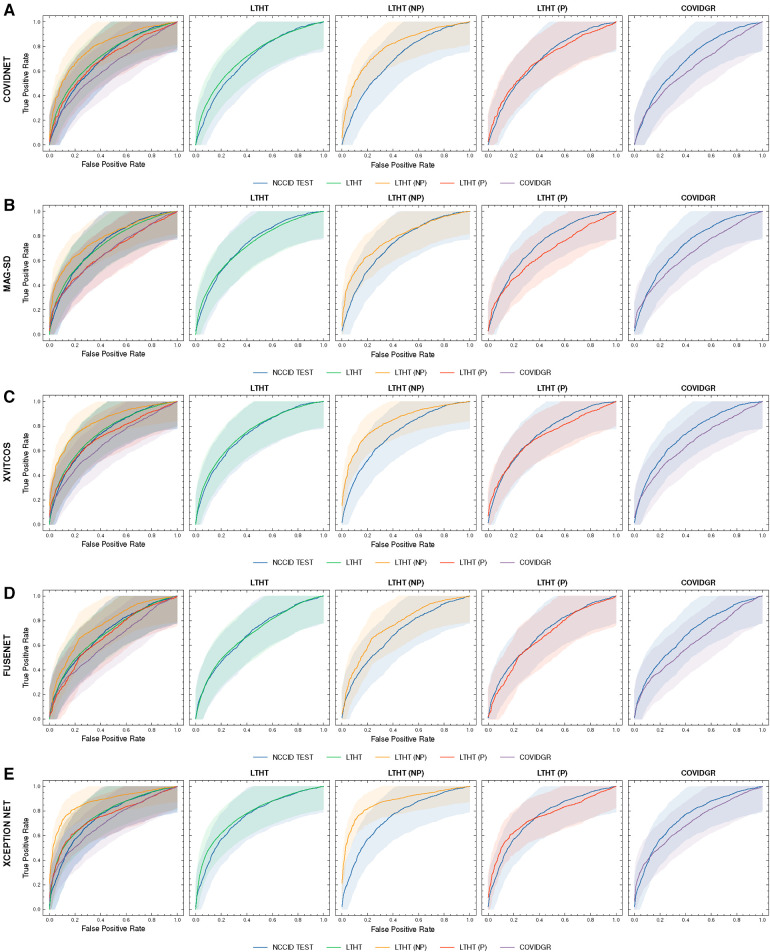
ROC curves of COVID-19 detection. Each row presents a different model, the first of each row presents ROC curves for all test data, the following columns present direct comparison between NCCID ROC curves and the dataset of interest, corresponding AUROC values can be found in Table 3. Shaded regions correspond to 95% confidence interval. (**A–E**) ROC curves of COVID-19 detection. (**F–J**) ROC curves of COVID-19 detection. ROC, Receiver Operating Characteristic; NCCID, National COVID-19 Chest Imaging Database; LTHT, Leeds Teaching Hospital Trust; AUROC, Area Under the Receiver Operating Characteristic.

Figure 4 shows a significant drop in performance when models are applied to an international dataset (COVIDGR). We found CORONET gives the most substantial decrease in performance, with model recall halving from LTHT (0.52) to COVIDGR (0.26). This decline in performance is further evidenced by a large drop in AUROC values from 0.70 in the national population (LTHT) to 0.60 in the international population (COVIDGR) (Table 3).

**Figure:**
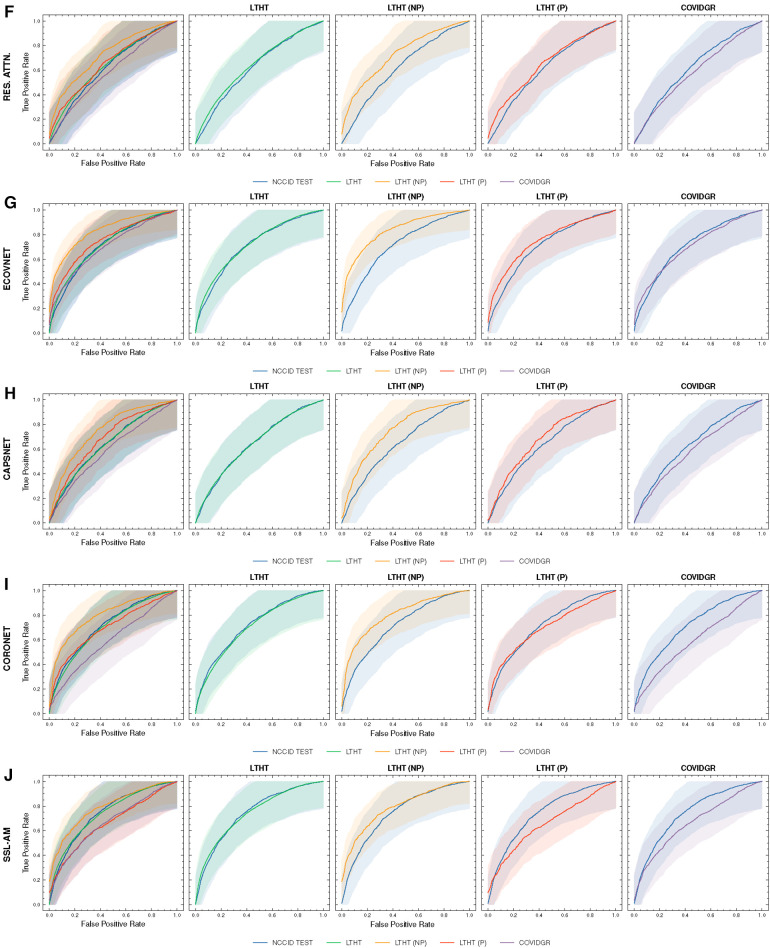


### 3.2 Model performance under counterfactual conditions

Our exploration of model performance in counterfactual scenarios gives insight into the impact of confounding disease features on COVID-19 detection i.e., non-COVID-19 pneumonia. In LTHT (NP) which removes other pneumonia types from the population, we observed clear improvements. All models achieve near perfect precision scores, ranging from 0.95 to 0.98, as well as substantially improved AUROC and recall scores (Figure 4 and Table 3). XCEPTION NET, the best-performing model in real-world LTHT cohorts, further improved with increases in AUROC scores from 0.78 to 0.88, recall scores from 0.65 to 0.73, and precision scores from 0.34 to 0.98 (Table 3).

When evaluating models under the alternative counterfactual scenario, using LTHT (P) where both COVID-19 and non-COVID-19 pneumonia are treated as the positive class i.e., models become general pneumonia classifiers, model performances diverge relative to performance on LTHT. Top-performing models in real-world data (LTHT) decrease in performance, as evidenced by especially reduced recall scores (Table 3). Of the best performing models we observe the greatest decline in AUROC in SSL-AM, from 0.74 (LTHT) to 0.66, suggesting that SSL-AM is best able to isolate features of COVID-19 pneumonia from non-COVID-19 pneumonia. The worst performing models (RES. ATTN., ECOVNET, CAPSNET and CORONET) all demonstrate improved performance on LTHT (P), suggesting that these were unable to learn to separate features of COVID-19 pneumonia from other pneumonia types.

When comparing top-performing models with their ROI-trained counterparts, the reduction in their performance under this counterfactual is greater. For example, XCEPTION NET (ROI) AUROC falls from 0.77 to 0.71, while the decrease in XCEPTION NET performance is less substantial. This indicates that ROI-trained models, compared to their full CXR trained counterparts, may have improved capacity for separating COVID-19 from non-COVID-19 pneumonia (Figure 9B).

### 3.3 Subgroup analysis

During sub-population analysis with independent NHS hospital data (LTHT), we observe disparities in model performance across demographic subgroups. We consider sex, ethnicity, age, smoking, and subgroups with comorbidities that are likely observable in a CXR. We report smoking and comorbidity analysis together due to overlap in clinical interest. Subgroups are described in detail in Section 2.

#### 3.3.1 Sex

**Figure 5:**
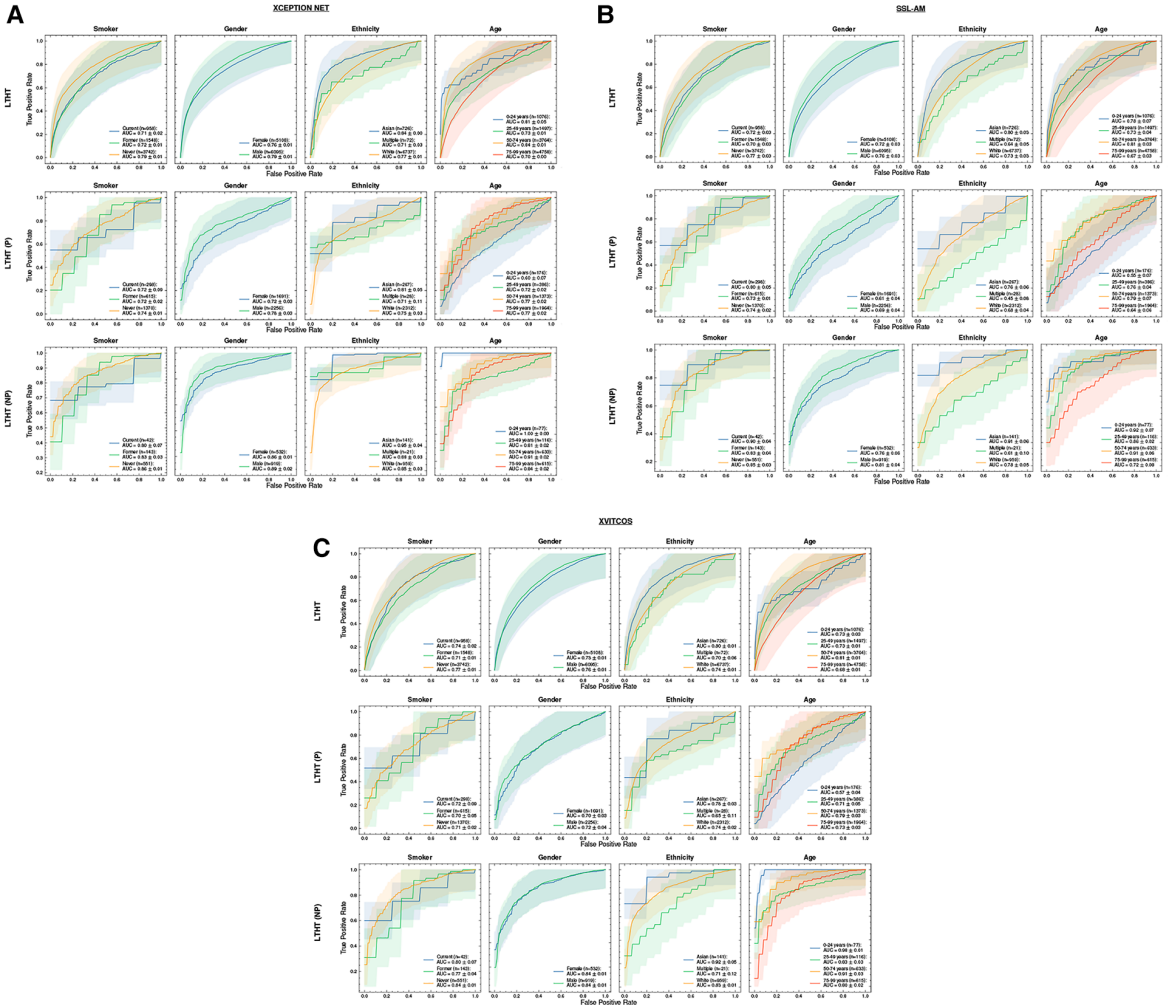
ROC curves of COVID-19 detection in smoker, sex, ethnicity and age subgroups for top-performing models: (**A**) XCEPTION NET, (**B**) SSL-AM, and (**C**) XVITCOS. The top row presents model performance in the real-world scenario and the bottoms rows present model performance under counterfactual conditions, LTHT (P) and LTHT (NP). Subgroup population size is referred to by *n*. Subgroups that do not exist in LTHT (P) or LTHT (NP) populations are excluded. Shaded regions correspond to 95% confidence intervals. (**A**) ROC curves of XCEPTION NET performance in smoker, sex, ethnicity and age subgroups. (**B**) ROC curves of SSL-AM performance in smoker, sex, ethnicity and age subgroups. (**C**) ROC curves of XVITCOS performance in smoker, sex, ethnicity and age subgroups. ROC, Receiver Operating Characteristic; AUC, Area Under Curve; LTHT, Leeds Teaching Hospital Trust.

**Figure 6:**
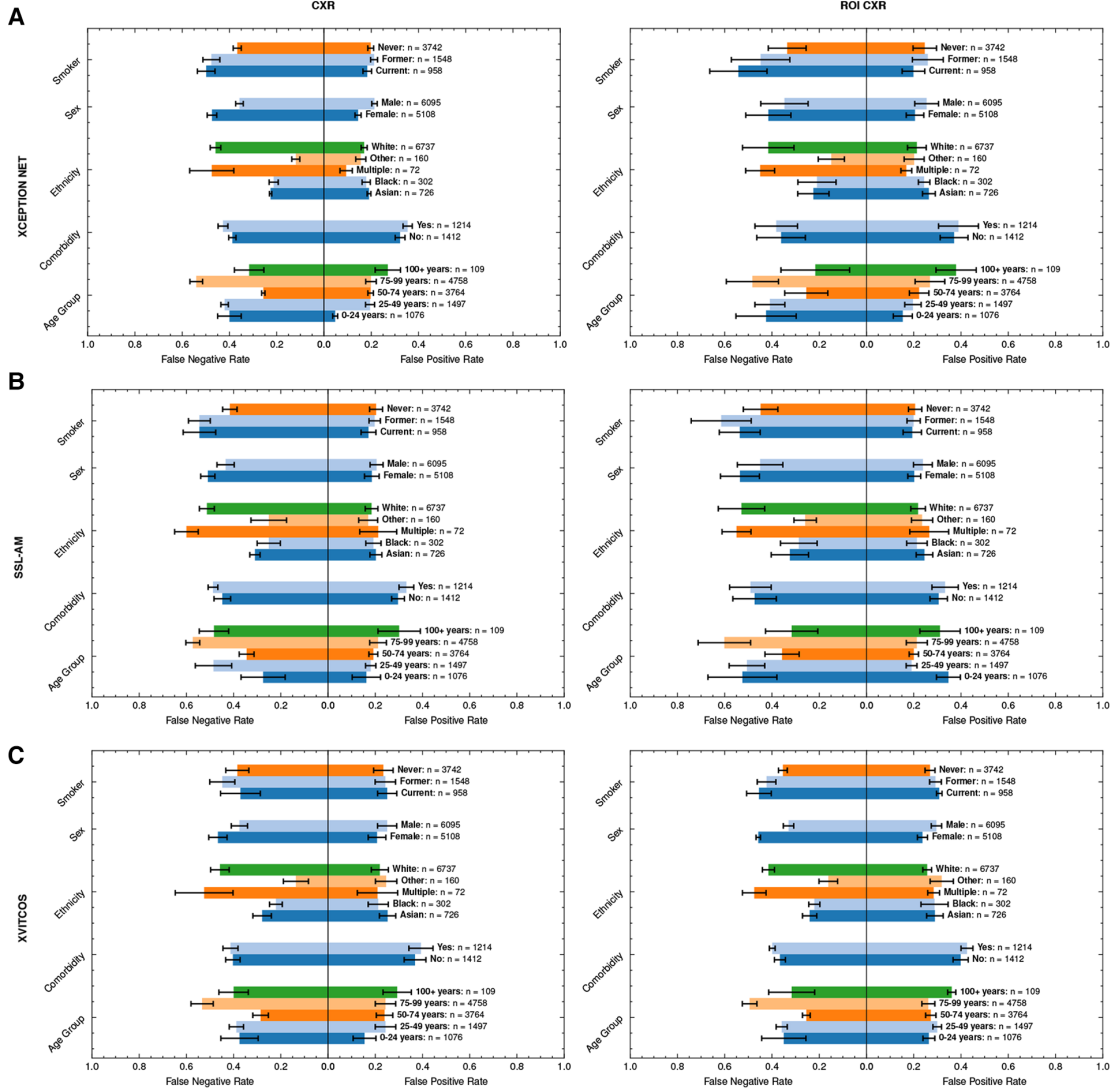
Average FPRs and FNRs of top-performing models, (**A**) XCEPTION NET, (**B**) SSL-AM, and (**C**) XVITCOS, and their ROI-trained counterparts in LTHT subgroups. Subgroup population size is referred to by *n*. Error bars correspond to standard deviation across cross-validations. FNR, False Negative Rate; FPR, False Positive Rate; ROI, Region of Interest; LTHT, Leeds Teaching Hospital Trust.

We found that, according to AUROC values, models perform better in male populations compared to female populations (Figure 5 and Supplementary Table S5). There is a consistent pattern of increased false negative predictions in the female population i.e., a greater proportion female COVID-19 cases are missed (Figure 6). Statistical significance in model AUROC disparities is confirmed in 5 out of 10 models (One-way ANOVA, p<0.05; Supplementary Table S6).

Sex bias persists even under counterfactual conditions; with the exception of XVITCOS, we observe this bias in models when applied to populations without alternative pneumonia types, LTHT(NP). This suggests that this bias cannot be due to differences in the prevalence of the non-COVID-19 pneumonia across the sexes. Upon further examination using real-world data (LTHT), we see that sex bias is not mitigated by ROI-extraction, the ROI-trained version of XVITCOS returns a higher rate of false negatives in the female population compared to the male population which is reflected in a larger recall scores in males (0.75) compared to females (0.62) (Supplementary Table S5 and Figure 6).

#### 3.3.2 Ethnicity

We also see statistically significant model performance disparities across ethnic subgroups (One-way ANOVA, p<0.05; Supplementary Table S6). XCEPTION NET AUROCs vary from 0.72 to 0.91 (Supplementary Table S5). All models appear to perform better when applied to Black and Asian groups, with significantly fewer false negatives. Supplementary Table S5 shows all models return higher precision score when applied to Black and Asian groups compared to White groups. XCEPTION NET precision falls from 0.73 (Black) and 0.52 (Asian) to 0.37 (White). This disparity of performance is also observed in the counterfactual without other pneumonia types, LTHT (NP). The performance gap between White, and Black and Asian groups, is unchanged with the use of ROI-trained models i.e., training on ROI CXRs has no effect.

#### 3.3.3 Age

We can observe similar statistically significant disparities in model performance across different age groups (One-way ANOVA, p<0.05; Supplementary Table S6). Generally, models perform best in the 50–74 age group, which is in line with COVID-19 prevalence by age group in the training data (Table 4). However, we observe that top-performing models appear more likely to return false negatives for the 75–99 age group, an age group at greater risk of adverse COVID-19 outcomes (Figure 6). Under counterfactual conditions, where all pneumonia types are included in the positive class, we see XCEPTION NET and XVITCOS models improve in performance in the 75–99 age group, indicating a reduced ability to separate COVID-19 pneumonia from non-COVID-19 pneumonia in older age groups (Figure 6). These comparisons should be interpreted cautiously, considering that the prevalence of non-COVID-19 pneumonia differs among subgroups.

**Table 4:** Demographic subgroups of training and test data.

	Sex (n)			Age	Ethnicity (n)					
	Male	Female	Unknown		Asian	Black	Multiple	Other	White	Unknown
NCCID										
Positive	61% (5,113)	38% (3,215)	0% (9)	67 ± 17	13% (1,120)	8% (739)	1% (118)	4% (379)	67% (5,592)	5% (389)
41% (8,337)										
Negative	58% (7,122)	42% (5,054)	0% (2)	70 ± 17	14% (1,687)	7% (913)	2% (230)	3% (421)	70% (559)	3% (3,680)
59% (12,178)										
N=20,515										
NCCID TEST										
Positive	71% (190)	29% (78)	0% (0)	66 ± 15	11% (29)	7% (19)	1% (4)	5% (13)	72% (192)	4% (11)
33% (268)										
Negative	64% (357)	36% (199)	0% (0)	69 ± 16	12% (67)	8% (42)	3% (14)	4% (20)	71% (392)	4% (21)
67% (556)										
N=824										
LTHT										
Positive	17% (1,061)	14% (691)	0% (0)	72 ± 16	10% (177)	7% (125)	1% (8)	3% (47)	67% (1,171)	13% (224)
16% (1,752)										
Negative	83% (5,034)	86% (4,417)	0% (1)	63 ± 26	6% (549)	2% (117)	1% (64)	1% (113)	59% (5,566)	32% (2,983)
84% (9,452)										
N=11,204										

Age is presented as mean ± standard deviation. Sex and ethnicity are presented both as absolute counts (n) and as percentages relative to the COVID-19 positive/negative cohort. NCCID, National COVID-19 Chest Imaging Database; LTHT, Leeds Teaching Hospital Trust; ROI, Region of Interest.

Figure 6 shows that models return the lowest false positive rate in the youngest age group i.e., 0–24 years, although this may be due to low prevalence of COVID-19 in this age group as indicated by combined low false positive rates and low precision scores (Supplementary Table S5). XVITCOS (ROI) gives improved performance in this age group compared to XVITCOS, with AUROC scores rising from 0.73 to 0.79. However, other ROI-trained models show a trend in reduced performance in this age group when compared to full CXR-trained counterparts, more so than other age groups (Figure 6). Comparison of SSL-AM and SSL-AM (ROI) shows a drastic fall in AUROC scores from 0.78 to 0.56 (Supplementary Table S5). Moreover, under the counterfactual condition in which models become general pneumonia classifiers, LTHT (P), we find that models perform particularly poorly in this age group (Figure 5); which could be interpreted as evidence of better separation of COVID-19 from non-COVID-19 pneumonia. We also observe that under LTHT (NP) conditions, with non-COVID-19 pneumonia removed, models perform better than in real-world populations of this subgroup (LTHT) e.g., XVITCOS AUROC scores increase from 0.73 to 0.96 (Figure 5).

#### 3.3.4 Smoking status & comorbidities

We observe a universal decline in model performance in the subgroup with CXR observable comorbidities. For 9 out of 10 models we evaluate statistically significant differences in model performance (AUROC) in these groups (One-way ANOVA, p<0.05; Supplementary Table S6). However, model performance on these subgroups could not be evaluated under counterfactual scenarios due to a lack of data.

In addition, we find that all models perform worse when applied to subgroups with a history of smoking (both former and current smokers) e.g., XCEPTION NET AUROC falls from 0.79 in subgroups without any smoking history to 0.73 and 0.51 in former and current smoker subgroups, respectively (Supplementary Table S5). We see increased false negative rates in these groups compared to non-smokers (Figure 6). Under counterfactual conditions where non-COVID-19 pneumonia is removed from the population, we see that model performance disparities between groups of different smoking status do not decrease. Models still perform best when applied to subgroups without any history of smoking, and performance disparities between former smoker and current smoker groups is sustained (Figure 5).

### 3.4 Model error analysis

**Figure 7:**
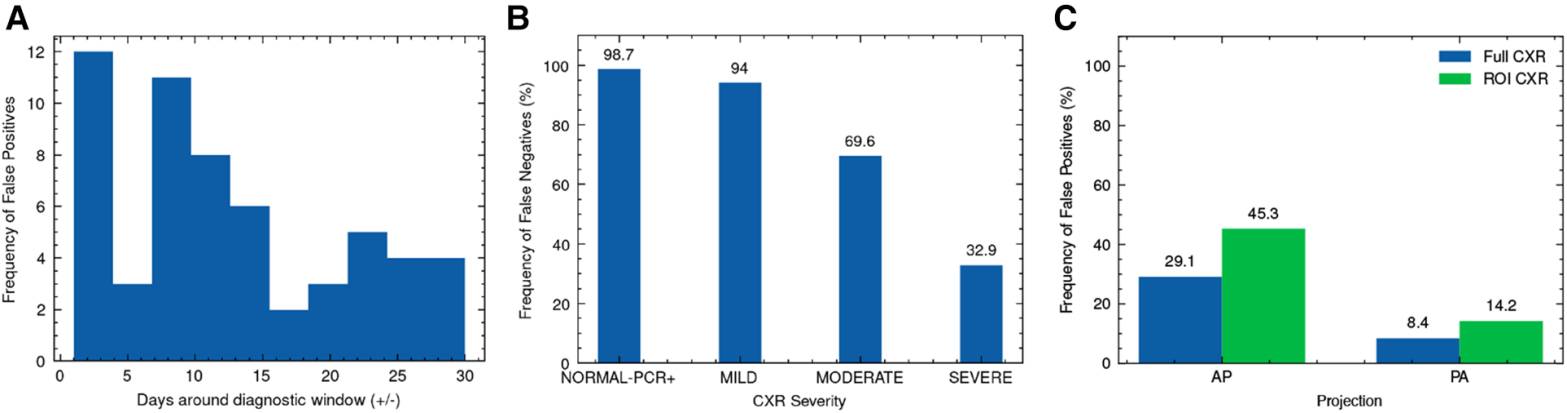
Analysis of XCEPTION NET prediction error. Frequency of (**A**) false positive predictions on NCCID TEST data according to proximity of CXR exam date to the diagnostic window, (**B**) false negative predictions on COVIDGR according to RALE-defined CXR severity and (**C**) false positive predictions on LTHT data according to CXR projection, alongside evaluation of ROI-trained XCEPTION NET false positive frequency. RALE, Radiographic Assessment of Lung Edema; ROI, Region of Interest; CXR, Chest x-ray; AP, Antero-posterior; PA, Postero-anterior.

We explore the influence of clinical and experimental factors on model error rate. As the best performing model, we used XCEPTION NET predictions for this analysis. During NCCID test population evaluation, we examined the relationship between the frequency of false positives and the recorded distance from CXR exam date to swab date-derived diagnostic window. According to this analysis, COVID-19 negative CXRs acquired in close proximity to the COVID-19 diagnostic window are more frequently predicted as COVID-19 compared to those obtained further away, with XCEPTION NET delivering the most false positives for CXRs 1-5 days before or after the diagnostic window (Figure 7A). We examine the correlation of incorrect COVIDGR predictions with radiologist-defined CXR severity. For this evaluation we characterised each CXR according to RALE criteria (21), labelling as “NORMAL-PCR+”, “MILD”, “MODERATE” and “SEVERE”. We observe a strong pattern of increasing frequency of false negative predictions for CXRs with milder features of COVID-19 disease i.e., MILD CXRs are more frequently missed. As expected, we find that 99% NORMAL-PCR+ CXRs are missed i.e., cases in which radiologists were unable to identify COVID-19 features. Even MILD and MODERATE COVID-19 CXRs exhibit high rates of false negatives, with COVID-19 being missed ≈94% and ≈70% of the time (Figure 7B). We find that CXRs categorised as SEVERE are less frequently missed, yet 32.9% are still falsely classed as COVID-19 negative.

With XCEPTION NET predictions, we evaluate the relationship between the projection view of the CXR and the frequency of false positive predictions. We found AP projected CXRs are more commonly misidentified as COVID-19 compared to PA projected CXRs (Figure 7C). Figure 7C also shows that ROI-trained XCEPTION NET makes more false positive predictions in AP projected CXRs than the full CXR trained XCEPTION NET.

### 3.5 Explainable AI highlights reliance on spurious correlations

**Figure 8:**
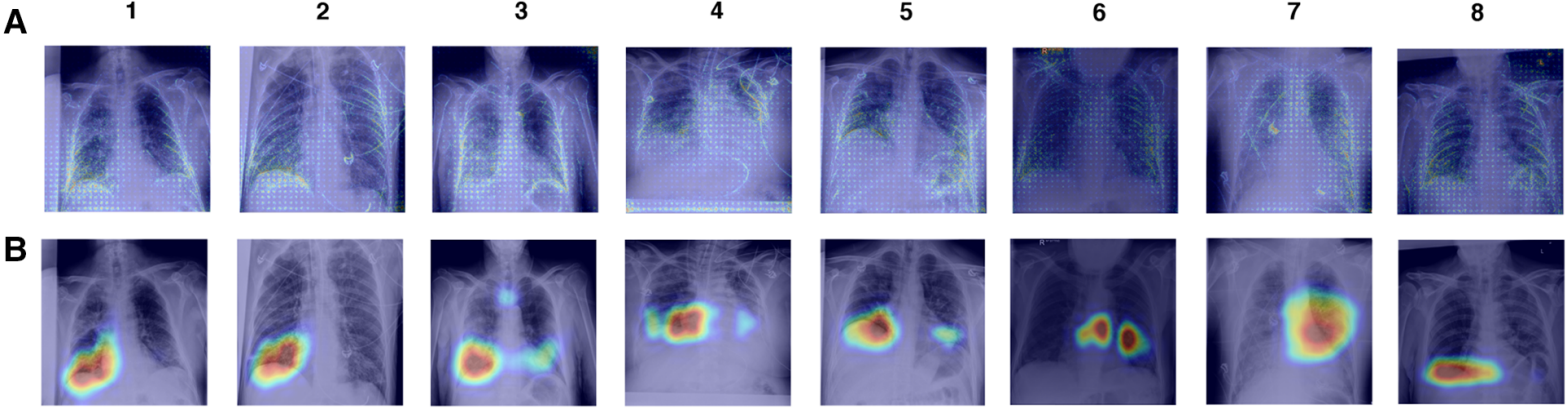
Saliency maps of correct XCEPTION NET predictions of COVID-19 positive CXRs. CXRs are sourced from the LTHT dataset and saliency maps are generated with (**A**) Gradient backpropagation and (**B**) GradCAM. LTHT, Leeds Teaching Hospital Trust; CXRs, Chest x-rays.

To investigate model reliance on spurious features, we create saliency maps using GradCAM and gradient backpropagation and examine the features that have the most influence on model predictions for both full CXR and ROI-trained models. We explore true positive COVID-19 predictions made by XCEPTION NET (Figure 8). Review of GradCAM saliency maps shows model reliance on both COVID-19 pathology and undesirable “shortcut” features. We found that clinically-relevant regions were consistently highlighted, with regions of significance often localised to the lower lung areas, as well as the heart margins of the cardiac silhouette. We observed similar activations in gradient backpropagation saliency maps. However, with this improved granularity we also observe highlighted support devices (i.e., heart monitor wiring, portacaths or endotracheal tubing) and radiograph annotations, possibly representing reliance on spurious “shortcut” features (Figure 8B).

### 3.6 Comparative validation of the impact of lung segmentation on model performance

**Figure 9:**
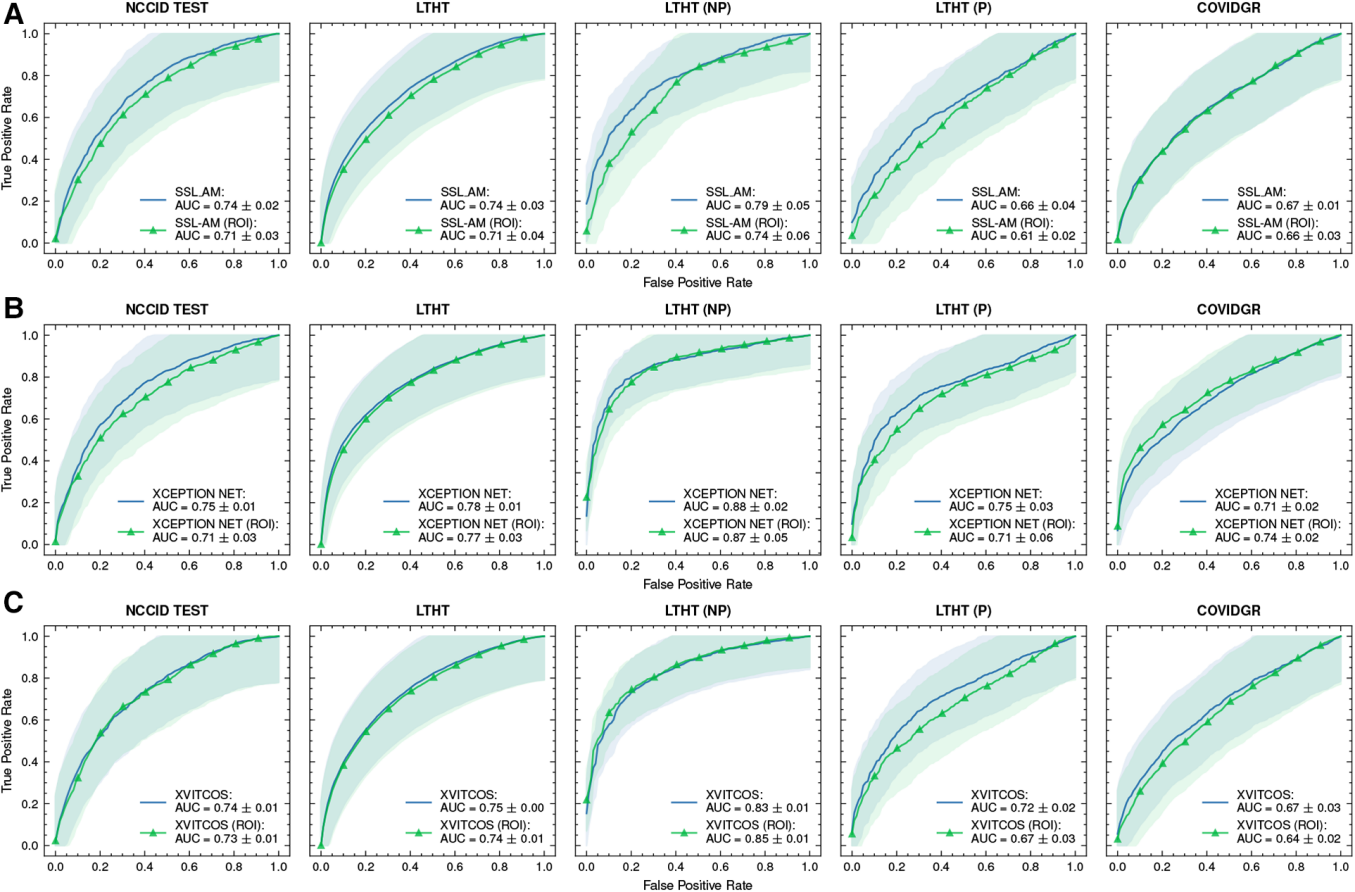
ROC curves of (**A**) SSL-AM, (**B**) XCEPTION NET, and (**C**) XVITCOS model evaluations on each dataset and their ROI-trained counterparts. Shaded regions correspond to the 95% confidence intervals. ROC, Receiver Operating Characteristic; AUC, Area Under Curve; ROI, Region of Interest.

We find that training models on ROI-extracted CXRs does not improve model performance. Against expectation, ROI-trained model performance is marginally worse compared to full CXR trained models when testing in NHS centre populations (LTHT). Notably, for XCEPTION NET and XVITCOS models, we find that training on ROI CXRs does not worsen performance in international population, instead performance marginally improves (Figure 9).

**Figure 10:**
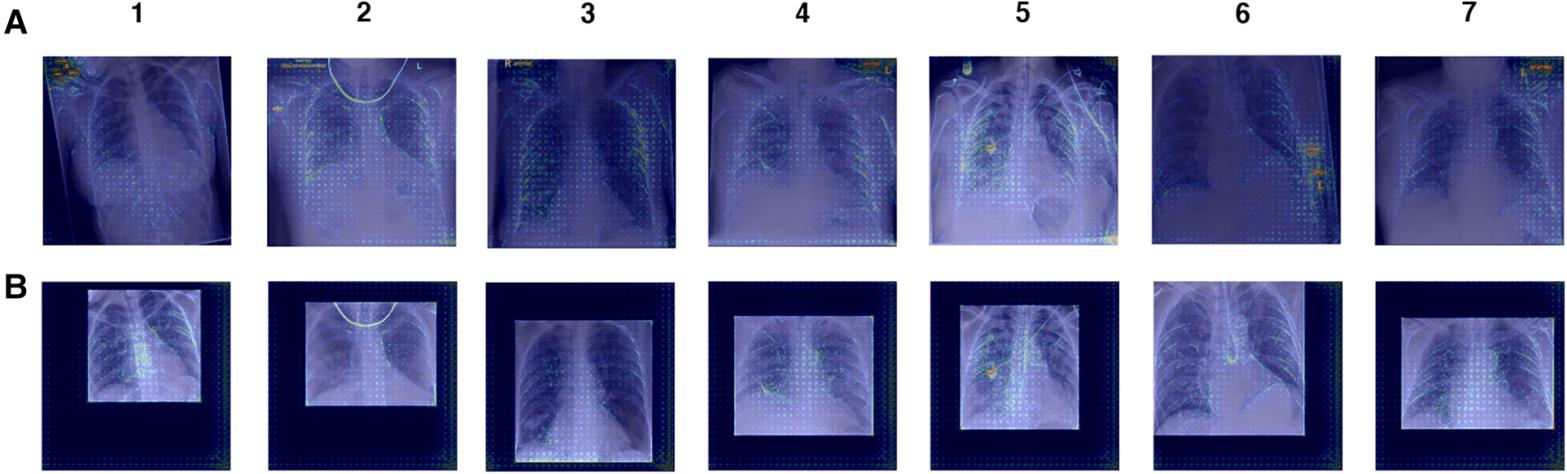
Gradient backpropagation feature attribution maps of true positive COVID-19 predictions. Saliency maps are generated for (**A**) XCEPTION NET predictions and (**B**) XCEPTION NET (ROI) predictions. XCEPTION NET (ROI) is trained and evaluated on CXRs cropped to the region of interest, while XCEPTION NET is trained on full CXRs. LTHT, Leeds Teaching Hospital Trust; CXRs, Chest x-rays; ROI, Region of Interest.

Manual inspection of the ROI-extracted CXRs showed that, with the use of a post-processing algorithm, lung regions were preserved (Supplementary Figure S4). Without loss of clinical features, we propose that the decrease in model performance is linked to the exclusion of non-clinical “shortcut” features, such as radiograph annotations, which have been identified in saliency maps of full CXR-trained models (Figure 8). These “shortcut” features are typically located outside the thoracic area, and are cropped out during ROI extraction (Supplementary Figure S4). However, while non-clinical features that exist outside the lung fields can be removed by cropping to the ROI, general health indicators e.g., presence of support devices, bone density, etc., remain in view and are highlighted in saliency maps, suggesting influence over model predictions (Figure 10).

Moreover, we find that ROI-trained models are worse at distinguishing COVID-19 from other pneumonia types, as evidenced by our evaluation of ROI-trained models in a subset of the LTHT populations with known pneumonia outcomes: LTHT (P) and LTHT (NP). ROI-trained models produce a much higher error rate in non-COVID-19 pneumonia populations. We observe that the ROI-trained version of XCEPTION NET performs much worse than its full CXR trained counterpart, with error rates of 0.51 compared to 0.40.

## 4 Discussion

The main goal of this research is to evaluate the use of deep learning approaches for the detection of COVID-19. We aim to identify the limitations of existing models to improve model readiness for implementation into clinical settings in the event of future sudden outbreaks.

When comparing our main findings with the existing literature we found that the model performances evaluated in this study contradict initial reports of model performance in many of the source publications, many of which report AUROC scores as exceeding 0.90. We identify several methodological flaws in this literature. Roberts et al. (4) considered 62 studies, including many of the studies selected for this benchmarking, and identified substantial limitations that placed the majority of models at high risk of bias (4). The main limitations considered by Roberts et al. (4) were the use of inappropriate training data, inadequate external validation and lack of subgroup evaluation (4). An additional critical analysis identifies that the data used in source publications put deep learning models at high risk of learning spurious “shortcuts” (5). Our retrospective study corrects these issues, with the use of multi-centre hospital data and extensive model validation on independent datasets. We report new findings in deep learning model performance and consider the major pitfalls in the development of deep learning models for clinical application.

Radiologists achieve performances of 0.78 AUROCS, as reported in (20). Although direct comparison between radiologist performance and model performance is inappropriate due to differences in test populations, at face-value deep learning models show promise as an assistive tool for use in future pandemics.

A comparison of model performance shows that the best performances on the real-world LTHT population are achieved by supervised deep CNN models that employ transfer learning. Pre-trained with ImageNet weights, top-performer XCEPTION NET can be characterised by the application of depth-wise separable convolutional operations for more efficient use of model parameters. The SSL-AM and XVITCOS models give the next strongest performances. SSL-AM is pre-trained with CheXpert data under self-supervised conditions to fully leverage the underlying data structures of a common domain. XVITCOS is a vision transformer, pre-trained on CheXpert, this approach uses positional embedding and self-attention to learn efficient CXR representations that incorporate both local features and global dependencies. In contrast, RES. ATTN. is the only model that does not apply pre-trained weights, and records the lowest performance metrics out of all evaluated models. From this we can speculate that transfer learning, domain-specific or otherwise, is needed to achieve strong model performance.

While classification metrics indicate adequate model performance, this study gives conclusive evidence that DL models perform poorly on clinically complex cases i.e., where comorbidities/confounding features are present, and frequently fail at separating COVID-19 from non-COVID-19 pneumonia. For example, decreased model performance is observed in populations with an increased incidence of clinically relevant underlying conditions e.g., the 74–99 age group, active/previous smokers, etc., these complex cases are disproportionately represented in hospital populations. Therefore, our findings suggest that existing COVID-19 detection models have limited value as an assistive tool for frontline radiologists, who are tasked with making challenging diagnoses for high risk patients that require urgent treatment.

These model failings can, in part, be attributed to inadequate data. In the absence of labels for alternative pathologies/classes supervised DL models are not equipped to learn to separate the similar features of different pathologies, e.g., non-COVID-19 pneumonia, emphysema, lung cancer, etc. Where pathologies co-occur more frequently with the class of interest than the negative class, models are vulnerable to blindly learning these features as “shortcuts” (5). Scarcity of multi-label datasets and widespread inadequacies in model validation highlight the need for clinicians and deep learning researchers to address the current shortfalls in data collection and to define criteria for clinically-oriented DL model development.

We tested models on the COVIDGR dataset to evaluate performance in international populations (Spain) where typical NHS clinical pathways and data acquisition protocols do not apply. Evaluation in COVIDGR shows that models generalise poorly outside NHS populations. However, in addition to changes in population characteristics, there are critical differences in how COVID-19 was defined. The diagnostic window we use to define COVID-19 cases in the NHS populations (−14/+28 days around RT-PCR+ swab) was decided under clinical guidance taking into consideration: the importance of early detection; poor RT-PCR sensitivity, particularly with low viral load as is observed in early stages of infection; and, typical time for CXR resolution post-infection. This is in stark contrast to COVIDGR which was pre-defined with a much shorter diagnostic window of 24 h before or after a positive RT-PCR swab. This raises the issue that without a standardised COVID-19 labelling protocol, which should balance technical feasibility with clinical utility, detection models are vulnerable to poor generalisability as a consequence of label shift. The likely use of open-source datasets in emergent pandemic situations, which can be compiled with inconsistent labelling as seen with COVID-19, highlights the importance of pro-active collaboration. For example, without clinical input deep learning researchers may unintentionally adopt a disease definition that maximises quantitative metrics i.e., accuracy, precision, recall etc., at the expense of clinical utility.

Moreover, we observe increased rates of false positive predictions in negative COVID-19 CXRs acquired close to the diagnostic window. With this evidence of increased diagnostic uncertainty and the understanding that, for a large portion of COVID-19 CXRs, disease features persist for a long time after infection, we suggest that current labelling strategies result in a noisy ground truth. A portion of post-COVID-19 resolved CXRs are either incorrectly considered COVID-19 or persistent disease features enter the control population. To reconcile this source of label noise, we propose the use of an additional category of COVID-19 disease which would separate chronic changes, i.e., persistent disease features post-infection, from active COVID-19 infection.

Additionally, the detection of COVID-19 through RT-PCR is flawed with low sensitivity and high rates of false negatives. Therefore, deriving COVID-19 status from RT-PCR testing alone adds further noise to the ground truth labels. In practice, the clinical diagnosis of COVID-19 takes into account more than just RT-PCR outcomes, e.g., clinical signs and symptoms, recent exposures, and patient medical history. In fact, 20% of symptomatic patients receive a clinical diagnosis of COVID-19 despite negative RT-PCR testing (22). We further propose a multi-modal labelling approach that would incorporate all relevant patient data, this would drastically reduce ground truth noise and benefit deep learning models, particularly supervised models.

In evaluating model performance awareness of bias and fairness is critical. Inadequate evaluation can allow biased deep learning models to amplify systemic healthcare disparities in under-served communities. Our evaluation of the models shows varied performance across different sub-populations, with top-performing models exhibiting obvious demographic biases, including unequal performance depending on ethnicity, sex and age. However, clinical evidence suggests that observed model performance disparities may be a consequence of varied disease severity between demographics. Generally, models perform better when applied to demographics which experience COVID-19 more severely, e.g., ethnic minorities, males, and older age groups (23, 24). The clinical factors affecting the severity of COVID-19 infection are still not fully understood. Before clinical implementation, a greater understanding is required to determine if these disparities in model performance might result in greater health inequity.

Crucially, our findings show that ROI-extraction was insufficient to prevent these disparities. Therefore, if bias is identified researchers should be cautioned against applying segmentation techniques with the assumption that the removal of background noise will fully mitigate the bias. Additionally, we find that cropping CXRs to the ROI prior to training does not improve overall model performance. This contradicts previous studies in which ROI-trained models performed better (25). A key difference between their approaches and ours is that we undertake a more rigorous methodology in which our segmentation model is trained on an external dataset. Whereas conflicting studies typically use the same data for both segmentation training and classification training, an approach that is not supportable in a clinical setting (25).

We evaluated the impact of CXR projection on model predictions, as recommended by Roberts et al. (4). AP projected CXRs are used when the patient is not able to get into the correct position for the standard PA projection, for example, if the patient is too ill or is in isolation (26). As a result, algorithms are at risk of learning to associate COVID-19 with projection rather than the clinically-relevant CXR features. We observe over-representation of AP CXRs in the disease class of the training data, 83% of positive COVID-19 images were AP projected, whilst only 65% of negative COVID-19 images were AP projected. Saliency maps provide evidence that projection may have been a spurious shortcut features, as they consistently highlight features around heart borders, a region of the CXR that varies greatly depending on projection. Moreover, AP CXRs predictions are more commonly false positive.

Our evaluations of a wide range of models suggests CXRs alone may not be sufficient to detect COVID-19. In a head-to-head comparison, performance metrics indicate that the top models are unable to compete with the gold standard clinical test, RT-PCR. Models often fail to separate COVID-19 from other pneumonia types and are unable to detect COVID-19 in RALE-defined NORMAL-PCR+ cases, in which 99% of COVID-19 positive CXRs are missed. Here, it is important to note that not all COVID-19 infections develop into COVID-19 pneumonia, in which case diagnostic features of COVID-19 cannot be observed in the CXR and even the very best DL models would be unable to detect COVID-19 infection.

In practice, it is rare for a disease diagnosis to be wholly determined by a single test. In fact, reducing the source of diagnostic information to a single modality risks losing diagnostic features of a disease. Where imaging is incongruous with patient health, clinicians often rely on additional sources of information. The incorporation of multi-modal information e.g., exposure data, symptoms, medical history, etc. during data curation should be more widely adopted to facilitate the development of improved DL models for the detection of COVID-19.

## 5 Conclusion

To conclude, clinical guidance is essential for the development of reliable predictive models, for disease diagnosis and medical image interpretation. In particular, we highlight the need for early and consistent disease definition, in order to ensure model generalisablity across international and jurisdictional populations. Disease definitions should also be continually reviewed for clinical utility, for instance, we suggest COVID-19 detection models could be improved by the separation of CXRs that exhibit long-term changes as a result of prior infection from CXRs of patients with active infection. To the extent that comparison is possible, the deep learning models evaluated detect COVID-19 with apparent similar performance to radiologists. However, both fall short of the gold standard clinical test, RT-PCR. Moreover, COVID-19 detection models have extreme difficulty identifying COVID-19 in complex clinical cases, as demonstrated by our evaluation of models in subgroups with higher incidences of confounding pathologies and comorbidities. Models are also vulnerable to learning “shortcut” features. Neither of these issues are mitigated by the use of lung segmentation. Ultimately, we suggest that a multi-modal approach under clinical guidance, where additional clinical factors are incorporated, is required to improve model performance; with the aim of developing a reliable assistive tool, on par with the existing gold standard, to help mass diagnosis in possible future outbreaks.

## Data Availability

The original contributions presented in the study are included in the article/**Supplementary Material**, further inquiries can be directed to the corresponding author.
