# Supplementary material for: Multi-centre benchmarking of deep learning models for COVID-19 detection in chest x-rays
Source: Front Radiol. 2024 May 21;4:1386906. doi: 10.3389/fradi.2024.1386906 (PMC11148230; doi:10.3389/fradi.2024.1386906)
Supplement: Supplementary file 1 [file Datasheet1.pdf]

Supplementary Material

The text, figures and tables below are supplementary to the article: *Multi-centre benchmarking of deep learning models for COVID-19 detection in chest x-rays*.

1 SUPPLEMENTARY DATA

1.1 Lung segmentation data (COVID-QU-Ex)

COVID-QU-Ex was released as a public COVID-19 dataset, the dataset comprises 11,956 COVID-19, 11,263 non-COVID-19 pneumonia, and 10,701 normal CXR images. In addition to chest X-rays (CXRs) and class labels, ground-truth lung masks were also included. Masks were generated by humans with machine assistance. Only frontal view CXRs were included, all images were either postero-anterior or antero-posterior.

Table S1. Inclusion and exclusion criteria of CXR exam from NCCID, LTHT and COVIDGR data. For all datasets, CXRs are eliminated if not frontal view. NCCID and LTHT CXR exams conducted after 2019 are eliminated if COVID-19 swab or image acquisition data is incomplete. For NCCID data, CXRs are also eliminated if submission centre data is incomplete or if the CXR exam date falls in between two non-overlapping windows of COVID-19 infection. *Abbrvs: National COVID-19 Chest Imaging Database (NCCID); Leeds Teaching Hospital Trust (LTHT); Antero-posterior (AP); Postero-anterior (PA); Chest X-ray (CXR).*

		**Data**					
		**NCCID**		**LTHT**		**COVIDGR**	
**Criteria**	Principle	*Pre-2019*	*Post-2019*	*Pre-2019*	*Post-2019*	*Pre-2019*	*Post-2019*
Inclusion	AP/PA CXRs	✓	✓	✓	✓	✓	✓
	Submission centre data	✓	✓				
	Complete swab data (data & outcome)		✓		✓		
	Complete image acquisition data (date & ID)	✓	✓	✓	✓		
Exclusion	Lateral or transverse CXRs	✓	✓	✓	✓	✓	✓
	LTHT submission centre	✓	✓				
	Exam data outside RT-PCR+swab window		✓				

1.2 Label generation

For NCCID and LTHT data, CXR labels were generated according to a pre-defined diagnostic window. Under clinical guidance, we defined the COVID-19 diagnostic window as 14 days before and 28 days after the acquisition data of a positive RT-PCR test swab. CXR exam date was evaluated relative to the nearest positive RT-PCR COVID-19 swab date, CXRs that fell inside this window (-14/+28 days) were labelled COVID-19 positive. In some cases, evaluation of serial patient swab dates created multiple non-overlapping diagnostic windows, we treated these as separate instances of COVID-19 infection and CXRs that fell between these windows were removed from the dataset. For COVIDGR, CXRs are provided with COVID-19 labels, COVID-19 CXRs are defined by a positive RT-PCR swab within 24 hours of CXR acquisition. We provide an illustration of the labelling schema through case by case examples (Supplementary Fig. S1).

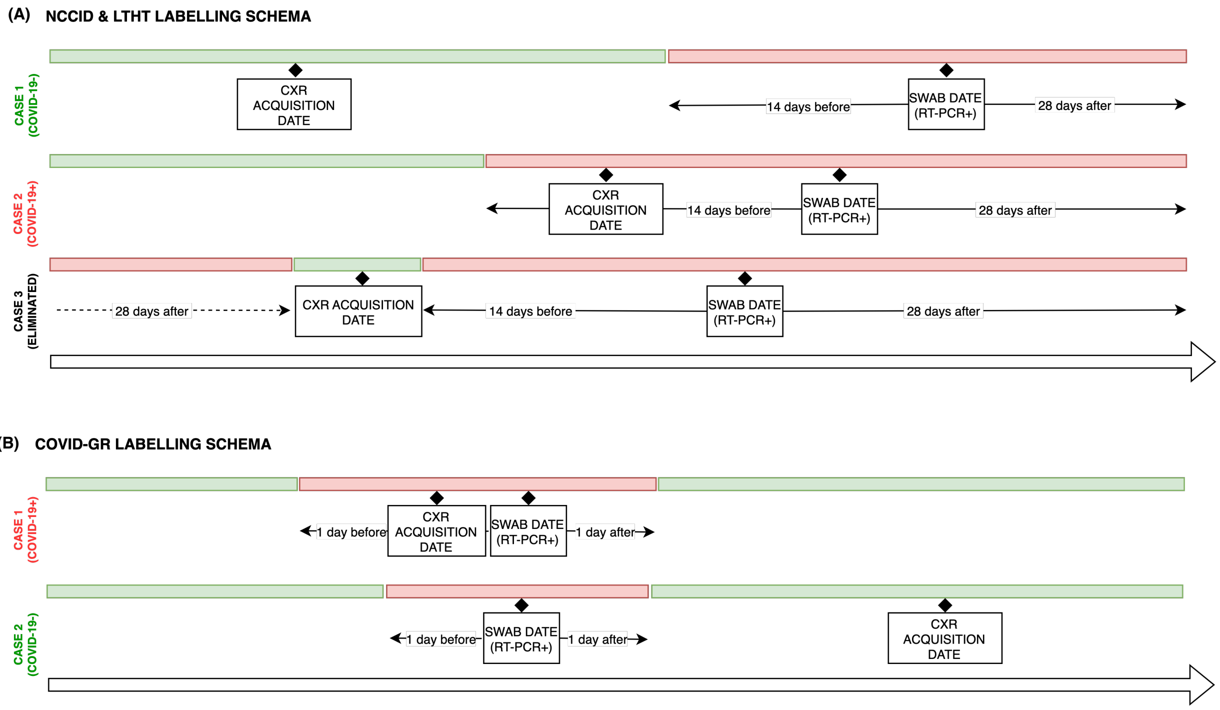

Figure S1: **Labelling schema for (A) NCCID and LTHT datasets, which share identical labelling protocol, and (B) COVIDGR data.** For each protocol we present examples of different label outcomes. **(A) CASE 1**: An illustration of CXR acquisition preceding the RT-PCR swab date diagnostic window (-14/+28 days), this case is therefore considered COVID-19 negative. **(A) CASE 2**: An example of CXR acquisition prior to the RT-PCR swab date but within the diagnostic window, as a result this case is labelled COVID-19 positive. **(A) CASE 3**: A scenario involving CXR elimination, where multiple swab tests are documented for a single case. If a CXR is acquired within the time frame between the windows around the swab dates, it is excluded from the dataset. **(B) CASE 1**: A case in which the CXR was acquired within the diagnostic window, specifically within 24 hours of the RT-PCR swab date (-1/+1 days). As a result, this case is designated as COVID-19 positive. **(B) CASE 2**: An example of CXR acquisition occurring after the diagnostic window, leading to the categorisation of this case as COVID-19 negative. *Abbrvs: Chest X-ray (CXR); National COVID-19 Chest Imaging Database (NCCID); Leeds Teaching Hospital Trust (LTHT); Reverse Transcription Polymerase Chain Reaction (RT-PCR).*

1.2.1 Counterfactual condition generation

Figure S2 show how CXR labels are combined to create the negative cohort and positive cohort that create the various counterfactual datasets.

1.2.2 Chest X-ray observable comorbidities

Comorbidities were categorised with clinical guidance. We grouped the LTHT population with recorded comorbidities into two categories: cases with comorbidities that could be observed in a CXR i.e., features of the disease are known to exist in the thoracic area, and cases without any CXR-observable comorbidities. Typically, the CXR-observable class of comorbidities comprises respiratory and cardiac diseases/disorders, whilst the non-observable class of comorbidities

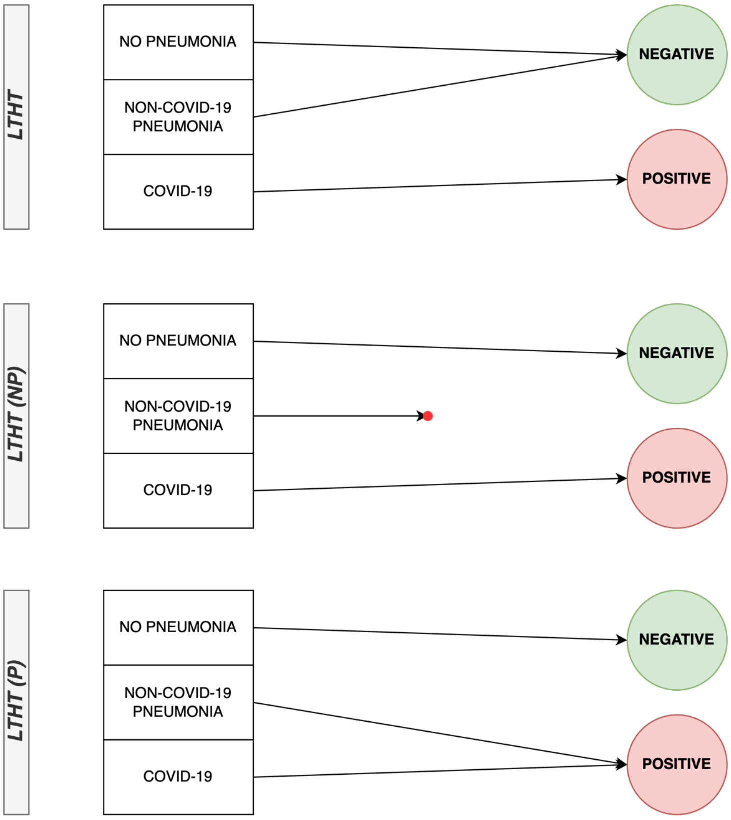

Figure S2: Generation of positive and negative cohorts of test dataset LTHT and counterfactual test datasets *LTHT NO PNEUMONIA (NP)* and *LTHT PNEUMONIA (P)*. The incomplete arrow indicates that on-COVID-19 pneumonia CXRs are not included in LTHT (NP). *Abbrvs: Leeds Teaching Hospital Trust (LTHT)*

comprises neurological diseases, inflammatory disorders (excluding respiratory), and blood diseases. Supplementary Table S2 presents an exhaustive list of the considered comorbidities and which category they belong to.

1.3 Image processing

All images were extracted from high resolution DICOM files and resized to 480x480 using area interpolation. To minimise risk of model overfitting a pipeline of image augmentations was applied, augmentations included: rotation, flipping, shifting, scaling, random brightness contrasting and random contrast. Image augmentations were applied uniformly during model training, with the exception of training under self-supervised conditions for which distortion, in-painting and perspective transformations were applied.

1.4 Model selection

We carried out an informal search for models of interest using a set of core keywords: ”chest X-rays”, “COVID-19” and (”deep learning” OR “artificial intelligence”). To ensure a wide variety of approaches were identified, we added deep learning-specific key words, these included, “anomaly detection”, “out-of-distribution”, “semi-supervised”, “weakly supervised”, “self-supervised”,

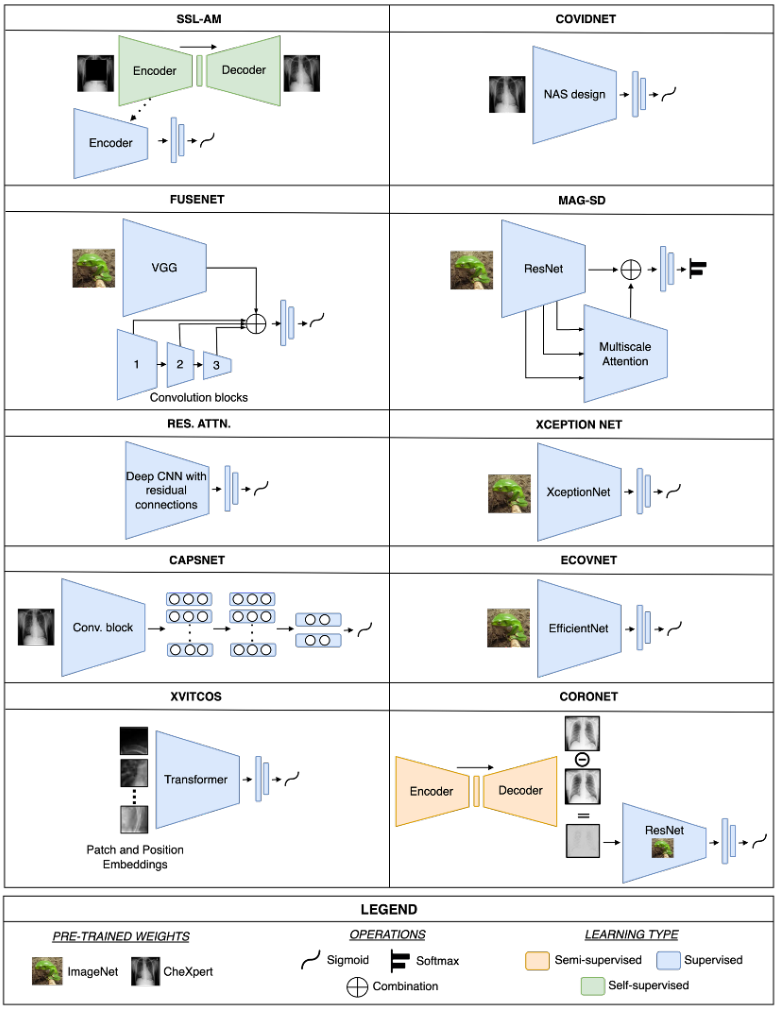

Figure S3: **Visual representation of evaluated models**. The applied CNN backbones are specified and colour-coding is used to indicate DL type. *Abbrvs: Deep Learning (DL).; Neural Architecture Search (NAS)*

Table S2. Categorisation of observed comorbidities. *Abbrvs: Chest X-ray (CXR); Chronic Obstructive Pulmonary Disease (COPD).*

CXR-observable	No CXR-observable
Cancer	Type 1 Diabetes
Cardiomyopathy	Type 2 Diabetes
Myocardial Infarction	Pancreatitis
Coronary Artery Disease	Liver Disease
Congestive Cardiac Failure	Gout
Hypertension	Dementia
COPD	Parkinsons Disease
Obesity	Motor Neurone Disease
Asthma	Other Neuromuscular Disease
Restrictive Lung Disease	Stroke
Other Respiratory Disorder	Thrombosis
	Transient Ischaemic Attack
	Peripheral Arterial Disease
	Rheumatoid Arthritis
	Ankylosing Spondylitis
	Peptic Ulcer Disease
	Paraplegia
	Spinal Injury

“unsupervised”, “generative”, “autoencoder”, and “uncertainty”. We searched an array of research databases, including, Google Scholar, PubMed, Scopus and IEEE Xplore. Considering the rapid development of this field and the importance of efficient dissemination of associated findings, pre-print manuscripts were intentionally included in the search.

1.5 Training protocol

We selected Binary Cross Entropy (BCE) as the objective function for all models with the exception of MAG-SD, for which we used the categorical cross entropy (due to model architecture restrictions). We tuned model hyperparameters via Optuna, an open source hyperparameter optimisation framework. The learning rates were tuned for pre-training and training stages. We searched learning rates from 0.01 to 0.0001, with intervals of 0.01. The learning rate that produced the lowest validation loss over 10 epochs was selected. All models are trained under 5-fold cross validation. During model training, if the validation loss plateaus for more than 5 epochs, learning rate is reduced by 10%. If validation loss does not improve for 15 epochs then model training is stopped.

1.6 Lung segmentation

The lung segmentation model was trained with a VGG backbone and UNet++ architecture. The VGG UNet++ was trained under deep supervision, where nested layer predictions inform model training. The VGG UNet++ was trained through minimisation of a soft DICE-BCE loss with the Adam optimiser algorithm. Learning rates were reduced by a factor of 0.8 with sustained plateaus in validation loss (10 epochs), and training was stopped if validation loss plateaued for more than 20 epochs. Qualitative evaluation of post-processed ROIs showed that this approach was largely successful in preserving all clinically-relevant anatomical structures in the CXR and eliminating ‘noise’, with consistent performance on both COVID-19 positive and COVID-19 negative CXRs (Supplementary Fig. S4).

(A)

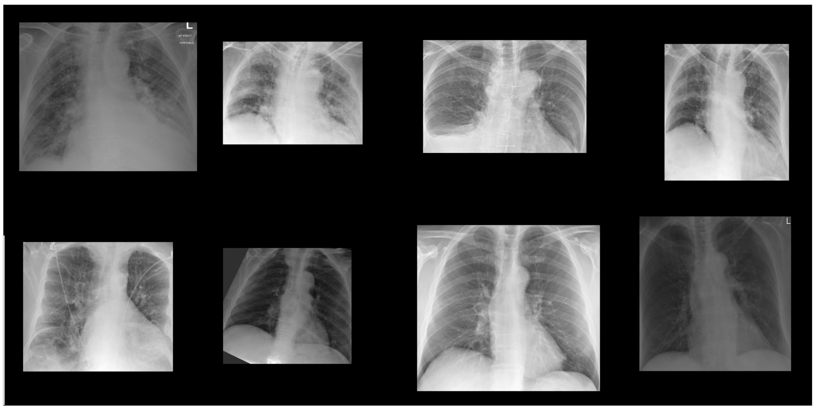

(B)

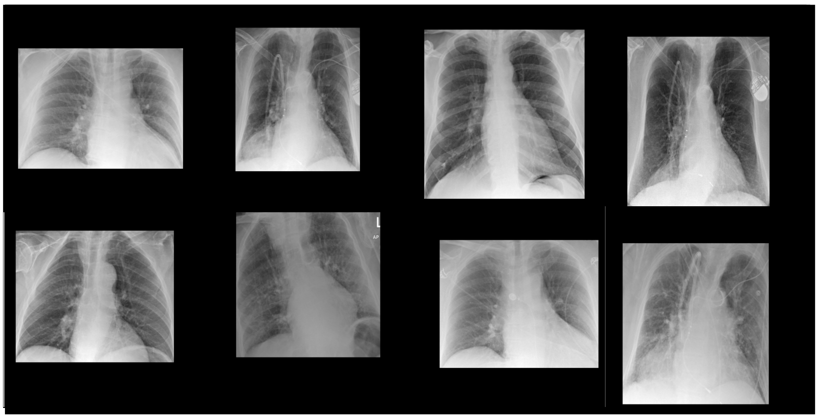

Figure S4: **ROI-extractions of post-processed semantic lung segmentations. (A)** Examples of ROI-extracted NCCID control cases. **(B)** ROI-extracted NCCID COVID-19 cases. *Abbrvs: Region Of Interest (ROI); National COVID-19 Chest Imaging Database (NCCID).*

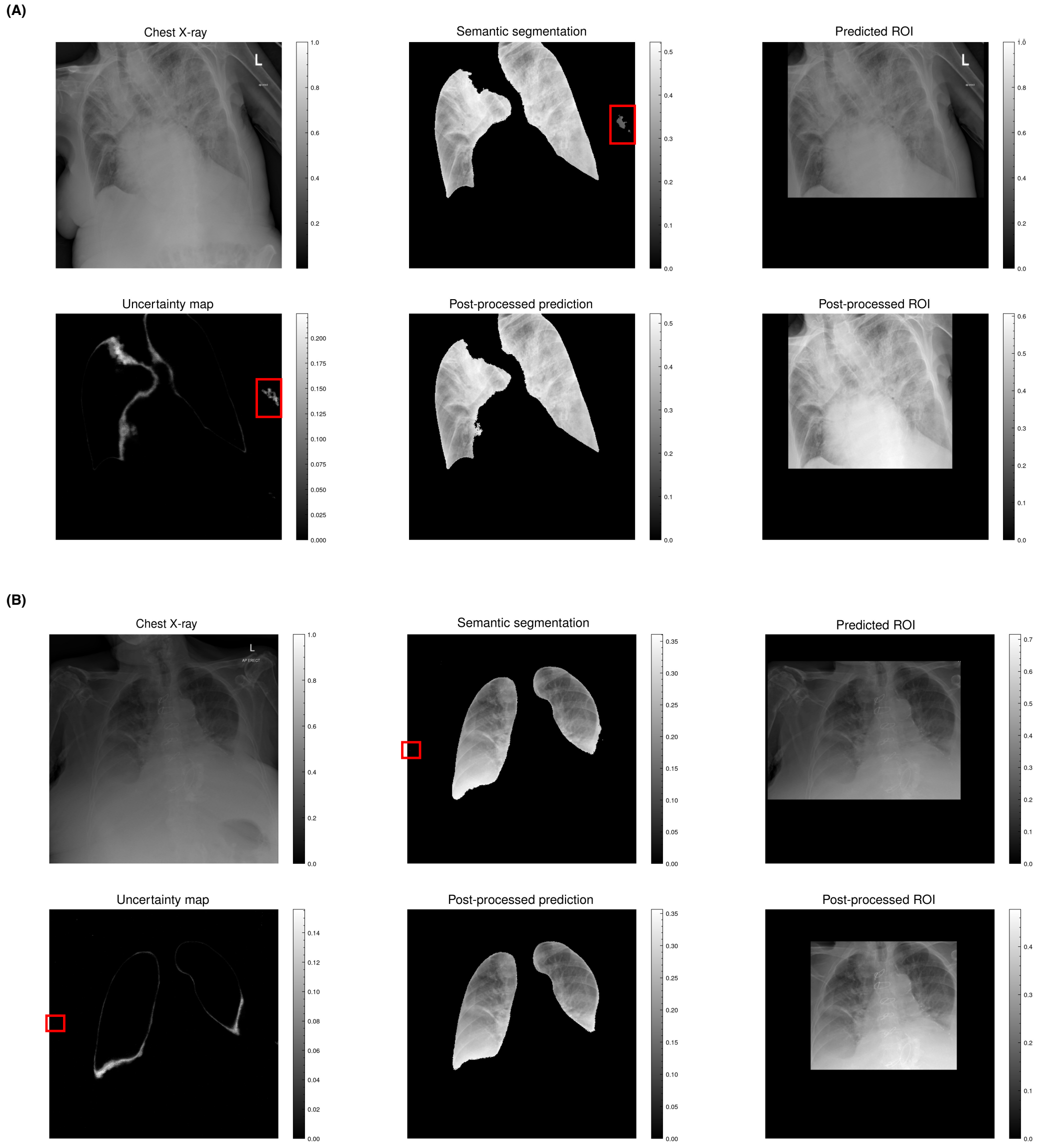

Figure S5: **Post-processing of COVID-19 CXR semantic lung segmentation to generate reliable ROIs. (A)** An example of severe COVID-19 in a CXR resulting in an uncertain prediction, with a total prediction uncertainty of 620. The right lung field is under-segmented, the additional structure is highlighted in the semantic segmentation and corresponding uncertainty map. This creates an overly large ROI around the lungs. Pixel-wise prediction uncertainty is used to isolate the extra structure, which is eliminated during post-processing of the semantic segmentation. **(B)** A COVID-19 CXR with under-segmentation of the lung fields (highlighted in the semantic segmentation and uncertainty map). Pixel-wise uncertainty is used to remove this structure from the semantic segmentation before creating a ROI. Total prediction uncertainty of this example is 230. *Abbrvs: Region Of Interest (ROI); Chest X-ray (CXR).*

2 SUPPLEMENTARY TABLES AND FIGURES

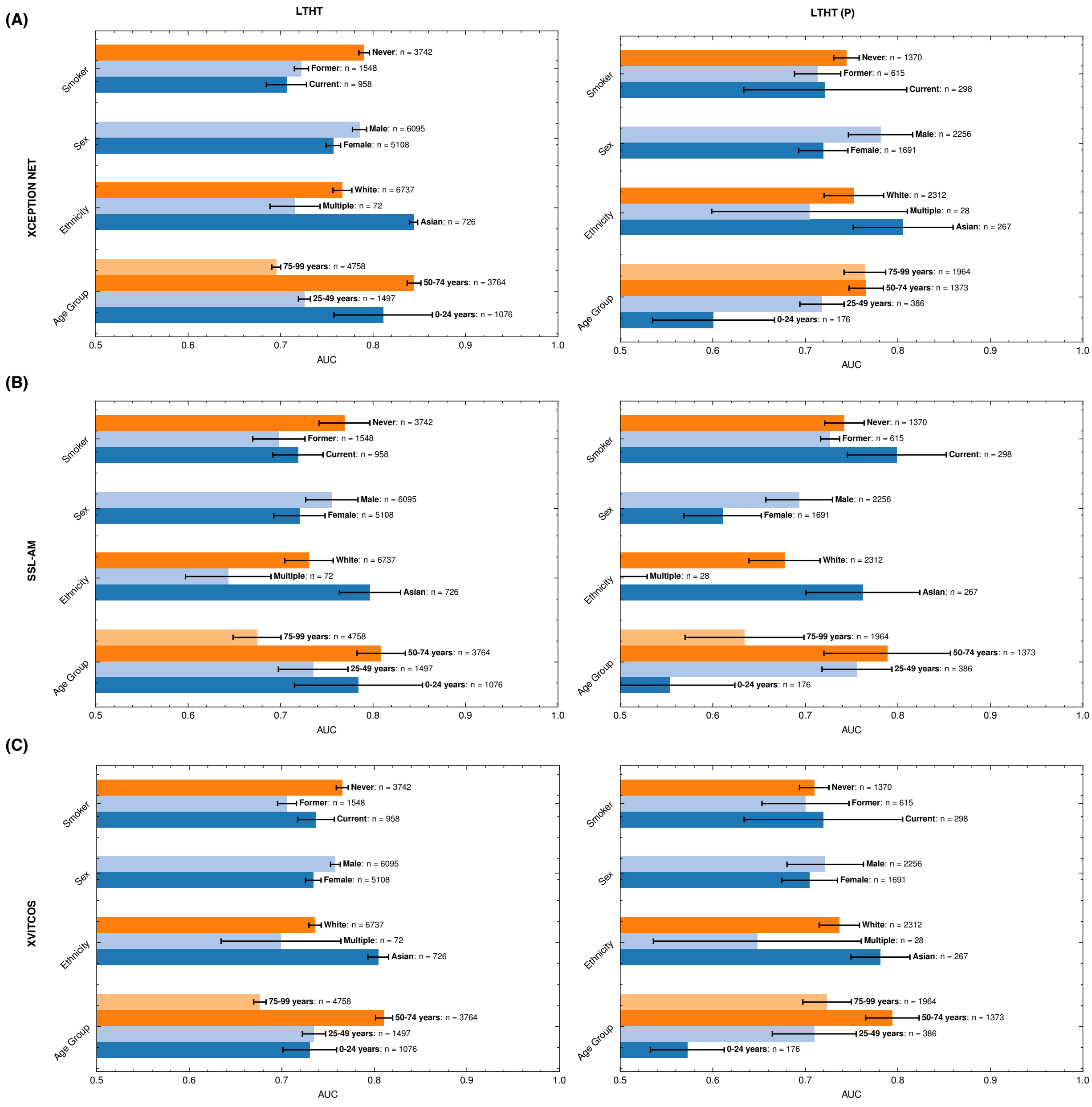

Figure S6: **(A) XCEPTION NET, (B) SSL-AM and (C) XVITCOS AUROCs for LTHT subgroup and LTHT (P) subgroups.** Only subgroups that exist in the LTHT (P) population are included. *n* is the subgroup population size. Error bars correspond to standard deviation across cross validations. *Abbrvs: Area Under Receiver Operator Characteristic (AUROC); Region of Interest (ROI).*

Table S3. Comparison of average full CXR-trained and ROI-trained model performance metrics. Standard deviation of metrics across cross-validation folds is included. *N* is the total number of cases in each test population. *Abbrvs: Chest X-ray (CXR); National COVID-19 Chest Imaging Database (NCCID); Leeds Teaching Hospital Trust (LTHT); Accuracy (Acc); Precision (Prec); Area Under the Receiver Operator Characteristic (AUROC).*

		**XCEPTION NET**		**SSL-AM**		**XVITCOS**	
		FULL	*ROI*	*FULL*	*ROI*	*FULL*	*ROI*
	Acc.	0.65 ± 0.01	0.60 ± 0.04	0.69 ± 0.03	0.61 ± 0.02	0.61 ± 0.03	0.61 ± 0.01
	Prec.	0.47 ± 0.01	0.44 ± 0.03	0.52 ± 0.03	0.44 ± 0.02	0.45 ± 0.02	0.45 ± 0.01
**NCCID TEST**	Recall	0.79 ± 0.01	0.76 ± 0.03	0.70 ± 0.05	0.75 ± 0.06	0.77 ± 0.02	0.77 ± 0.02
	F1	0.59 ± 0.01	0.55 ± 0.02	0.59 ± 0.01	0.56 ± 0.02	0.57 ± 0.01	0.56 ± 0.01
	AUROC	0.75 ± 0.01	0.71 ± 0.03	0.74 ± 0.02	0.71 ± 0.03	0.74 ± 0.01	0.73 ± 0.01
N=824							
	Acc.	0.75 ± 0.01	0.66 ± 0.04	0.72 ± 0.03	0.68 ± 0.02	0.70 ± 0.03	0.66 ± 0.02
	Prec.	0.34 ± 0.01	0.28 ± 0.02	0.30 ± 0.03	0.27 ± 0.01	0.30 ± 0.02	0.27 ± 0.01
**LTHT**	Recall	0.65 ± 0.02	0.74 ± 0.02	0.62 ± 0.03	0.60 ± 0.08	0.66 ± 0.04	0.69 ± 0.02
	F1	0.45 ± 0.01	0.41 ± 0.03	0.41 ± 0.03	0.37 ± 0.02	0.41 ± 0.01	0.39 ± 0.01
	AUROC	0.78 ± 0.01	0.77 ± 0.03	0.74 ± 0.03	0.71 ± 0.04	0.75 ± 0.01	0.74 ± 0.01
N=11,204							
	Acc.	0.75 ± 0.02	0.87 ± 0.01	0.69 ± 0.03	0.73 ± 0.05	0.74 ± 0.03	0.84 ± 0.02
	Prec.	0.98 ± 0.00	0.96 ± 0.02	0.96 ± 0.01	0.95 ± 0.01	0.97 ± 0.01	0.95 ± 0.00
**LTHT (NP)**	Recall	0.73 ± 0.02	0.90 ± 0.02	0.68 ± 0.03	0.75 ± 0.06	0.73 ± 0.04	0.87 ± 0.03
	F1	0.84 ± 0.01	0.93 ± 0.01	0.80 ± 0.03	0.83 ± 0.04	0.83 ± 0.02	0.91 ± 0.01
	AUROC	0.88 ± 0.02	0.87 ± 0.05	0.79 ± 0.05	0.74 ± 0.06	0.83 ± 0.01	0.85 ± 0.01
N=3,948							
	Acc.	0.52 ± 0.02	0.64 ± 0.03	0.49 ± 0.02	0.50 ± 0.06	0.55 ± 0.04	0.58 ± 0.02
	Prec.	0.99 ±. 0.00	0.98 ± 0.01	0.98 ± 0.00	0.98 ± 0.00	0.99 ± 0.00	0.98 ± 0.00
**LTHT (P)**	Recall	0.50 ± 0.02	0.64 ± 0.03	0.48 ± 0.03	0.50 ± 0.07	0.54 ± 0.04	0.58 ± 0.03
	F1	0.67 ± 0.02	0.77 ± 0.02	0.65 ± 0.02	0.66 ± 0.05	0.70 ± 0.04	0.73 ± 0.02
	AUROC	0.75 ± 0.03	0.71 ± 0.06	0.66 ± 0.04	0.61 ± 0.02	0.72 ± 0.02	0.67 ± 0.03
N=1,451							
	Acc.	0.62 ± 0.01	0.65 ± 0.02	0.59 ± 0.03	0.58 ± 0.03	0.61 ± 0.02	0.59 ± 0.01
	Prec.	0.86 ± 0.05	0.91 ± 0.01	0.78 ± 0.05	0.77 ± 0.05	0.73 ± 0.04	0.69 ± 0.02
**COVIDGR**	Recall	0.30 ± 0.02	0.33 ± 0.04	0.26 ± 0.10	0.23 ± 0.05	0.34 ± 0.04	0.33 ± 0.03
	F1	0.44 ± 0.02	0.49 ± 0.04	0.38 ± 0.10	0.35 ± 0.07	0.46 ± 0.04	0.44 ± 0.02
	AUROC	0.71 ± 0.02	0.74 ± 0.02	0.67 ± 0.01	0.66 ± 0.03	0.67 ± 0.03	0.64 ± 0.02
N=852							

Table S4: **Multiple (pair-wise) model performance comparisons with Tukey's Honest Significant Difference (HSD) test.** *Abbrvs: Accuracy (Acc.); Precision (Prec.); Area Under the Receiver Operator Characteristic (AUROC); XCEPTION NET (XCEPTION); Adjusted p-value (p-adj).*

		**Acc.**		**AUROC**		**F1**		**Prec.**		**Recall**	
Group 1	*Group 2*	*mean diff.*	*p-adj*	*mean diff.*	*p-adj*	*mean diff.*	*p-adj*	*mean diff.*	*p-adj*	*mean diff.*	*p-adj*
CAPSNET	CORONET	0.0550	0.6572	0.0571	0.0308	0.0314	0.8277	0.0416	0.4585	-0.0005	1.0000
CAPSNET	COVIDNET	0.0165	0.9999	0.0779	0.0008	0.0607	0.0814	0.039	0.5505	0.1243	0.3164
CAPSNET	ECOVNET	0.0862	0.1107	0.0748	0.0014	0.0684	0.0307	0.0767	0.0067	0.0046	1.0000
CAPSNET	FUSENET	-0.0127	1.0000	0.0499	0.0913	0.0342	0.7488	0.0195	0.9873	0.1039	0.5636
CAPSNET	MAG-SD	0.0223	0.9985	0.0818	0.0004	0.0630	0.0616	0.0426	0.4272	0.1174	0.3940
CAPSNET	RES. ATTN.	-0.0101	1.0000	-0.0000	1.0000	-0.0006	1.0000	-0.0051	1.0	0.0176	1.0000
CAPSNET	SSL-AM	0.0533	0.6936	0.0961	0.0000	0.0816	0.0047	0.0643	0.0405	0.1019	0.5889
CAPSNET	XCEPTION	0.0895	0.0856	0.1301	0.0000	0.125	0.0000	0.1056	0.0001	0.1316	0.2456
CAPSNET	XVITCOS	0.0360	0.9576	0.1019	0.0000	0.0822	0.0043	0.0565	0.1085	0.1426	0.1613
CORONET	COVIDNET	-0.0385	0.9366	0.0208	0.9503	0.0293	0.8772	-0.0027	1.0000	0.1248	0.3117
CORONET	ECOVNET	0.0312	0.9832	0.0177	0.9821	0.0370	0.6591	0.0351	0.6843	0.0050	1.0000
CORONET	FUSENET	-0.0677	0.3741	-0.0072	1.0000	0.0028	1.0000	-0.0221	0.9715	0.1043	0.5576
CORONET	MAG-SD	-0.0327	0.9770	0.0247	0.8705	0.0316	0.8226	0.0009	1.0000	0.1178	0.3886
CORONET	RES. ATTN.	-0.0650	0.4296	-0.0571	0.0307	-0.0321	0.8107	-0.0468	0.3006	0.0180	1.0000
CORONET	SSL-AM	-0.0016	1.0000	0.0390	0.3431	0.0502	0.2530	0.0226	0.9665	0.1024	0.5830
CORONET	XCEPTION	0.0345	0.9676	0.0730	0.0019	0.0935	0.0007	0.0640	0.0421	0.1321	0.2416
CORONET	XVITCOS	-0.0190	0.9996	0.0448	0.1793	0.0508	0.2382	0.0148	0.9983	0.1430	0.1583
COVIDNET	ECOVNET	0.0697	0.3349	-0.0031	1.0000	0.0077	1.0000	0.0378	0.5921	-0.1197	0.3663
COVIDNET	FUSENET	-0.0292	0.9893	-0.0280	0.7680	-0.0265	0.9285	-0.0194	0.9879	-0.0204	1.0000
COVIDNET	MAG-SD	0.0058	1.0000	0.0039	1.0000	0.0023	1.0000	0.0036	1.0000	-0.0070	1.0000
COVIDNET	RES. ATTN.	-0.0265	0.9946	-0.0779	0.0008	-0.0614	0.0753	-0.0441	0.3784	-0.1067	0.5264
COVIDNET	SSL-AM	0.0369	0.9509	0.0182	0.9786	0.0208	0.9842	0.0253	0.9345	-0.0224	1.0000
COVIDNET	XCEPTION	0.0730	0.2759	0.0523	0.0648	0.0642	0.0531	0.0666	0.0293	0.0073	1.0000
COVIDNET	XVITCOS	0.0195	0.9995	0.0240	0.8893	0.0215	0.9806	0.0175	0.9942	0.0183	1.0000
ECOVNET	FUSENET	-0.0989	0.0387	-0.0249	0.8649	-0.0342	0.7487	-0.0572	0.0994	0.0993	0.6232
ECOVNET	MAG-SD	-0.0639	0.4552	0.0070	1.0000	-0.0054	1.0000	-0.0342	0.7157	0.1128	0.4493
ECOVNET	RES. ATTN.	-0.0962	0.0488	-0.0748	0.0014	-0.0691	0.0282	-0.0819	0.0030	0.0130	1.0000
ECOVNET	SSL-AM	-0.0328	0.9764	0.0213	0.9432	0.0131	0.9995	-0.0125	0.9996	0.0974	0.6482
ECOVNET	XCEPTION	0.0033	1.0000	0.0553	0.0406	0.0565	0.1320	0.0289	0.8669	0.1271	0.2885
ECOVNET	XVITCOS	-0.0502	0.7606	0.0271	0.8007	0.0138	0.9993	-0.0203	0.9837	0.1380	0.1933
FUSENET	MAG-SD	0.0350	0.9642	0.0319	0.6163	0.0288	0.8877	0.0230	0.9629	0.0135	1.0000
FUSENET	RES. ATTN.	0.0027	1.0000	-0.0499	0.0910	-0.0349	0.7289	-0.0247	0.9435	-0.0863	0.7820
FUSENET	SSL-AM	0.0661	0.4080	0.0462	0.1498	0.0474	0.3249	0.0447	0.3591	-0.0019	1.0000
FUSENET	XCEPTION	0.1022	0.0289	0.0803	0.0005	0.0907	0.0012	0.0861	0.0016	0.0277	0.9999
FUSENET	XVITCOS	0.0487	0.7887	0.0520	0.0673	0.0480	0.3075	0.0369	0.6224	0.0387	0.9987
MAG-SD	RES. ATTN.	-0.0324	0.9785	-0.0818	0.0004	-0.0637	0.0568	-0.0477	0.2756	-0.0998	0.6172
MAG-SD	SSL-AM	0.0310	0.9837	0.0143	0.9962	0.0185	0.9930	0.0217	0.9744	-0.0154	1.0000
MAG-SD	XCEPTION	0.0672	0.3854	0.0483	0.1131	0.0619	0.0705	0.0630	0.0477	0.0143	1.0000
MAG-SD	XVITCOS	0.0137	1.0000	0.0200	0.9602	0.0192	0.9911	0.0139	0.999	0.0252	1.0000
RES. ATTN.	SSL-AM	0.0634	0.4654	0.0961	0.0000	0.0822	0.0043	0.0694	0.0199	0.0844	0.8029
RES. ATTN.	XCEPTION	0.0995	0.0366	0.1302	0.0000	0.1256	0.0000	0.1107	0.0000	0.114	0.4338
RES. ATTN.	XVITCOS	0.0461	0.8367	0.1019	0.0000	0.0829	0.0039	0.0616	0.0576	0.1250	0.3093
SSL-AM	XCEPTION	0.0361	0.9568	0.0341	0.5297	0.0434	0.4448	0.0413	0.4683	0.0297	0.9998
SSL-AM	XVITCOS	-0.0173	0.9998	0.0058	1.0000	0.0006	1.0000	-0.0078	1.0000	0.0406	0.9981
XCEPTION	XVITCOS	-0.0534	0.6913	-0.0283	0.7586	-0.0428	0.4655	-0.0491	0.2398	0.0110	1.0000

Table S5: **Average model performance metrics for each subgroup in the LTHT population.** Standard deviation of metrics across cross-validation folds is included. **Bold** indicates the highest average metric per dataset. *Abbrvs: National COVID-19 Chest Imaging Database (NCCID); Leeds Teaching Hospital Trust (LTHT); Accuracy (Acc); Precision (Prec); Area Under Curve (AUC); Female (F); Male (M); XCEPTION NET (XCEPTION).*

		**Ethnicity**					**Age Group**					**CXR Comorbidity**		**Smoking Status**			**Sex**	
Subgroups		*White*	*Black*	*Asian*	*Multiple*	*Other*	*0-24*	*25-49*	*50-74*	*75-99*	*100+*	*Y*	*N*	*Never*	*Former*	*Current*	*M*	*F*
**COVIDNET**	*Acc.*	0.68±0.04	0.74±0.01	0.71±0.04	0.68±0.07	0.73±0.05	0.75±0.08	0.68±0.04	0.70±0.03	0.65±0.04	0.68±0.05	0.59±0.00	0.63±0.02	0.68±0.04	0.66±0.04	0.68±0.04	0.63±0.02	0.59±0.01
	Prec.	0.30±0.03	0.66±0.05	0.46±0.05	0.17±0.04	0.54±0.06	0.03±0.01	0.17±0.02	0.39±0.03	0.25±0.02	0.20±0.04	0.62±0.01	0.72±0.01	0.31±0.03	0.18±0.02	0.13±0.01	0.72±0.01	0.64±0.01
	Recall	0.60±0.05	0.81±0.06	0.76±0.05	0.48±0.15	0.84±0.05	0.80±0.10	0.68±0.06	0.77±0.04	0.51±0.06	0.63±0.14	0.62±0.01	0.65±0.06	0.68±0.05	0.57±0.05	0.59±0.11	0.68±0.05	0.58±0.05
	F1	0.40±0.01	0.72±0.12	0.57±0.01	0.25±0.06	0.65±0.04	0.05±0.01	0.28±0.01	0.51±0.01	0.33±0.00	0.30±0.05	0.62±0.02	0.68±0.03	0.42±0.02	0.28±0.01	0.21±0.02	0.70±0.03	0.61±0.03
	AUC	0.71±0.01	0.85±0.01	0.80±0.01	0.58±0.10	0.86±0.02	0.84±0.03	0.74±0.01	0.80±0.00	0.63±0.01	0.71±0.04	0.61±0.01	0.66±0.01	0.75±0.00	0.65±0.01	0.69±0.03	0.65±0.00	0.62±0.01
**MAG-SD**	*Acc.*	0.69±0.04	0.72±0.03	0.71±0.04	0.65±0.17	0.75±0.07	0.69±0.16	0.68±0.06	0.73±0.03	0.65±0.04	0.58±0.09	0.59±0.02	0.62±0.02	0.70±0.03	0.67±0.04	0.68±0.06	0.63±0.02	0.58±0.03
	Prec.	0.30±0.03	0.63±0.03	0.46±0.04	0.20±0.09	0.56±0.09	0.03±0.01	0.18±0.03	0.41±0.03	0.26±0.03	0.22±0.06	0.63±0.02	0.72±0.01	0.32±0.03	0.18±0.02	0.13±0.02	0.73±0.02	0.63±0.02
	Recall	0.59±0.07	0.80±0.03	0.75±0.04	0.53±0.05	0.83±0.04	0.83±0.06	0.68±0.06	0.73±0.03	0.54±0.09	0.55±0.21	0.61±0.06	0.64±0.06	0.67±0.05	0.54±0.04	0.59±0.05	0.68±0.06	0.56±0.06
	F1	0.40±0.02	0.71±0.02	0.57±0.03	0.28±0.10	0.67±0.07	0.05±0.03	0.28±0.04	0.52±0.02	0.35±0.03	0.22±0.06	0.62±0.04	0.68±0.03	0.43±0.02	0.27±0.02	0.21±0.02	0.70±0.03	0.59±0.04
	AUC	0.71±0.02	0.81±0.02	0.79±0.03	0.62±0.12	0.86±0.05	0.86±0.09	0.74±0.04	0.80±0.01	0.66±0.04	0.61±0.08	0.63±0.02	0.66±0.02	0.76±0.02	0.67±0.02	0.69±0.03	0.65±0.02	0.62±0.03
**XVITCOS**	*Acc.*	0.70±0.03	0.77±0.02	0.70±0.03	0.71±0.08	0.76±0.03	0.78±0.06	0.69±0.04	0.72±0.03	0.67±0.03	0.64±0.07	0.60±0.01	0.63±0.01	0.70±0.03	0.68±0.04	0.69±0.03	0.64±0.00	0.59±0.01
	Prec.	0.32±0.02	0.69±0.03	0.44±0.02	0.22±0.06	0.56±0.04	0.02±0.01	0.18±0.01	0.40±0.03	0.27±0.01	0.19±0.04	0.63±0.01	0.71±0.01	0.32±0.02	0.21±0.01	0.15±0.01	0.73±0.01	0.64±0.01
	Recall	0.62±0.04	0.81±0.03	0.77±0.02	0.60±0.15	0.90±0.04	0.63±0.08	0.66±0.05	0.77±0.03	0.55±0.05	0.63±0.07	0.66±0.03	0.67±0.04	0.68±0.04	0.62±0.06	0.69±0.08	0.70±0.03	0.60±0.04
	F1	0.42±0.01	0.75±0.02	0.56±0.01	0.32±0.06	0.69±0.03	0.04±0.01	0.28±0.02	0.53±0.02	0.29±0.05	0.29±0.05	0.64±0.01	0.69±0.02	0.43±0.02	0.31±0.01	0.24±0.01	0.71±0.01	0.62±0.02
	AUC	0.74±0.01	0.85±0.01	0.80±0.01	0.70±0.06	0.87±0.01	0.73±0.03	0.73±0.01	0.81±0.01	0.68±0.01	0.70±0.05	0.63±0.01	0.66±0.01	0.77±0.01	0.71±0.01	0.74±0.02	0.65±0.01	0.64±0.01
**XVITCOS (ROI)**	*Acc.*	0.67±0.01	0.71±0.04	0.68±0.03	0.65±0.03	0.70±0.03	0.64±0.03	0.63±0.02	0.69±0.02	0.65±0.02	0.59±0.04	0.61±0.01	0.64±0.01	0.67±0.02	0.63±0.02	0.62±0.02	0.64±0.01	0.59±0.01
	Prec.	0.30±0.01	0.62±0.04	0.42±0.03	0.18±0.02	0.49±0.03	0.01±0.00	0.15±0.01	0.38±0.01	0.27±0.01	0.17±0.02	0.62±0.01	0.71±0.01	0.30±0.01	0.18±0.01	0.12±0.01	0.71±0.01	0.64±0.01
	Recall	0.67±0.03	0.82±0.02	0.81±0.03	0.63±0.08	0.88±0.04	0.70±0.06	0.68±0.03	0.80±0.02	0.59±0.03	0.73±0.08	0.68±0.02	0.70±0.02	0.71±0.03	0.64±0.04	0.66±0.01	0.75±0.02	0.62±0.02
	F1	0.41±0.00	0.70±0.03	0.55±0.02	0.28±0.03	0.63±0.03	0.03±0.00	0.25±0.01	0.52±0.01	0.37±0.01	0.28±0.04	0.65±0.01	0.71±0.01	0.42±0.01	0.28±0.01	0.20±0.01	0.73±0.01	0.63±0.01
	AUC	0.73±0.01	0.82±0.02	0.80±0.02	0.68±0.05	0.85±0.03	0.79±0.04	0.72±0.01	0.81±0.01	0.68±0.01	0.68±0.05	0.64±0.01	0.66±0.02	0.76±0.01	0.70±0.02	0.69±0.01	0.64±0.01	0.63±0.01
**FUSENET**	*Acc.*	0.65±0.07	0.70±0.04	0.68±0.05	0.75±0.01	0.71±0.04	0.69±0.09	0.65±0.10	0.67±0.07	0.62±0.07	0.65±0.09	0.57±0.01	0.61±0.03	0.65±0.07	0.64±0.01	0.65±0.10	0.60±0.03	0.57±0.02
	Prec.	0.28±0.03	0.62±0.06	0.42±0.09	0.25±0.06	0.51±0.05	0.02±0.00	0.15±0.02	0.37±0.05	0.24±0.02	0.16±0.04	0.60±0.01	0.70±0.03	0.28±0.04	0.17±0.02	0.12±0.02	0.70±0.03	0.63±0.02
	Recall	0.59±0.10	0.77±0.07	0.73±0.02	0.50±0.16	0.80±0.08	0.68±0.06	0.60±0.12	0.71±0.09	0.50±0.10	0.50±0.17	0.60±0.09	0.64±0.10	0.65±0.09	0.55±0.09	0.59±0.11	0.66±0.10	0.56±0.10
	F1	0.37±0.01	0.31±0.04	0.53±0.02	0.31±0.04	0.62±0.03	0.03±0.01	0.24±0.01	0.48±0.03	0.33±0.02	0.23±0.06	0.60±0.05	0.66±0.05	0.39±0.02	0.26±0.02	0.20±0.02	0.67±0.05	0.59±0.05
	AUC	0.68±0.01	0.79±0.02	0.77±0.04	0.64±0.01	0.82±0.02	0.75±0.12	0.68±0.01	0.76±0.01	0.63±0.01	0.60±0.05	0.59±0.01	0.63±0.02	0.71±0.01	0.64±0.01	0.67±0.03	0.61±0.01	0.60±0.01
**XCEPTION**	*Acc.*	0.75±0.01	0.80±0.01	0.77±0.01	0.87±0.02	0.84±0.02	0.93±0.02	0.75±0.02	0.76±0.01	0.71±0.02	0.69±0.05	0.61±0.01	0.65±0.01	0.74±0.01	0.72±0.01	0.75±0.02	0.64±0.01	0.62±0.00
	Prec.	0.37±0.01	0.73±0.02	0.52±0.01	0.43±0.09	0.68±0.01	0.06±0.01	0.20±0.01	0.46±0.01	0.30±0.00	0.23±0.04	0.65±0.01	0.74±0.01	0.36±0.01	0.22±0.01	0.16±0.01	0.73±0.01	0.68±0.01
	Recall	0.60±0.03	0.80±0.02	0.80±0.00	0.53±0.09	0.89±0.01	0.63±0.00	0.60±0.02	0.78±0.01	0.53±0.03	0.78±0.11	0.62±0.02	0.67±0.02	0.68±0.02	0.59±0.03	0.55±0.06	0.69±0.02	0.58±0.02
	F1	0.46±0.01	0.76±0.01	0.63±0.01	0.47±0.08	0.77±0.02	0.12±0.02	0.30±0.01	0.57±0.01	0.38±0.01	0.36±0.05	0.63±0.01	0.70±0.01	0.47±0.01	0.32±0.01	0.24±0.02	0.71±0.01	0.63±0.01
	AUC	0.77±0.01	0.86±0.00	0.84±0.00	0.72±0.03	0.91±0.01	0.81±0.01	0.73±0.01	0.84±0.01	0.70±0.00	0.77±0.04	0.65±0.01	0.69±0.01	0.79±0.01	0.73±0.01	0.51±0.02	0.66±0.00	0.66±0.01
**XCEPTION (ROI)**	*Acc.*	0.67±0.04	0.74±0.03	0.67±0.06	0.70±0.11	0.73±0.07	0.68±0.15	0.69±0.04	0.70±0.03	0.61±0.03	0.52±0.06	0.62±0.01	0.67±0.01	0.65±0.03	0.63±0.03	0.68±0.02	0.66±0.01	0.63±0.01
	Prec.	0.31±0.03	0.64±0.04	0.42±0.05	0.24±0.10	0.54±0.08	0.02±0.01	0.19±0.02	0.40±0.03	0.26±0.01	0.17±0.02	0.63±0.01	0.72±0.01	0.30±0.02	0.19±0.01	0.13±0.01	0.72±0.01	0.66±0.01
	Recall	0.72±0.03	0.87±0.03	0.83±0.01	0.65±0.15	0.88±0.03	0.73±0.05	0.68±0.03	0.83±0.02	0.66±0.04	0.87±0.04	0.72±0.03	0.76±0.01	0.77±0.03	0.68±0.03	0.62±0.05	0.76±0.03	0.71±0.03
	F1	0.43±0.03	0.74±0..02	0.56±0.04	0.35±0.12	0.66±0.06	0.04±0.02	0.29±0.02	0.54±0.02	0.37±0.01	0.29±0.03	0.67±0.01	0.73±0.01	0.43±0.02	0.29±0.02	0.22±0.02	0.74±0.01	0.68±0.01
	AUC	0.76±0.03	0.86±0.03	0.81±0.04	0.74±0.08	0.89±0.03	0.77±0.09	0.76±0.01	0.83±0.02	0.69±0.02	0.76±0.04	0.66±0.01	0.68±0.01	0.78±0.02	0.71±0.03	0.71±0.03	0.66±0.01	0.67±0.01
**RES. ATTN.**	*Acc.*	0.66±0.04	0.68±0.02	0.65±0.03	0.73±0.05	0.64±0.01	0.72±0.07	0.67±0.03	0.66±0.03	0.63±0.05	0.63±0.09	0.55±0.02	0.57±0.03	0.65±0.04	0.66±0.05	0.68±0.04	0.55±0.02	0.56±0.03
	Prec.	0.26±0.02	0.60±0.01	0.37±0.03	0.60±0.01	0.42±0.02	0.02±0.01	0.15±0.02	0.33±0.02	0.22±0.02	0.17±0.06	0.60±0.02	0.70±0.02	0.26±0.02	0.16±0.01	0.10±0.01	0.70±0.02	0.62±0.02
	Recall	0.50±0.06	0.67±0.10	0.64±0.11	0.60±0.05	0.60±0.05	0.58±0.20	0.55±0.09	0.62±0.08	0.45±0.07	0.63±0.07	0.52±0.07	0.54±0.07	0.57±0.08	0.45±0.08	0.46±0.04	0.53±0.06	0.53±0.09
	F1	0.34±0.02	0.63±0.05	0.47±0.04	0.34±0.04	0.49±0.01	0.03±0.01	0.23±0.03	0.42±0.02	0.29±0.02	0.26±0.07	0.55±0.04	0.61±0.04	0.35±0.02	0.23±0.02	0.17±0.01	0.60±0.04	0.57±0.05
	AUC	0.64±0.02	0.75±0.05	0.70±0.03	0.65±0.04	0.69±0.03	0.70±0.08	0.66±0.06	0.70±0.02	0.58±0.02	0.63±0.07	0.57±0.02	0.61±0.02	0.66±0.03	0.61±0.02	0.61±0.02	0.58±0.01	0.59±0.02
**ECOVNET**	*Acc.*	0.75±0.01	0.78±0.02	0.76±0.02	0.90±0.03	0.84±0.01	0.92±0.02	0.81±0.00	0.76±0.01	0.68±0.02	0.61±0.05	0.57±0.01	0.59±0.02	0.73±0.03	0.73±0.01	0.80±0.01	0.60±0.02	0.56±0.01
	Prec.	0.34±0.02	0.73±0.02	0.50±0.03	0.62±0.24	0.69±0.01	0.04±0.01	0.23±0.02	0.45±0.02	0.24±0.01	0.18±0.02	0.64±0.00	0.74±0.02	0.33±0.02	0.18±0.02	0.16±0.02	0.74±0.01	0.65±0.01
	Recall	0.47±0.03	0.73±0.05	0.65±0.06	0.35±0.05	0.83±0.03	0.40±0.15	0.45±0.07	0.65±0.05	0.41±0.03	0.68±0.10	0.51±0.03	0.54±0.04	0.56±0.04	0.40±0.05	0.42±0.04	0.57±0.04	0.45±0.03
	F1	0.39±0.02	0.73±0.03	0.56±0.04	0.44±0.09	0.75±0.01	0.07±0.03	0.30±0.03	0.53±0.03	0.31±0.02	0.28±0.03	0.56±0.02	0.62±0.03	0.41±0.03	0.25±0.03	0.23±0.02	0.64±0.03	0.53±0.03
	AUC	0.71±0.02	0.84±0.02	0.77±0.03	0.67±0.05	0.88±0.01	0.77±0.05	0.71±0.02	0.79±0.03	0.62±0.02	0.69±0.04	0.61±0.01	0.65±0.02	0.73±0.03	0.63±0.03	0.68±0.02	0.64±0.01	0.61±0.02
**CAPSNET**	*Acc.*	0.67±0.04	0.69±0.03	0.66±0.04	0.66±0.09	0.66±0.04	0.80±0.06	0.64±0.07	0.67±0.03	0.63±0.04	0.63±0.07	0.56±0.01	0.55±0.02	0.65±0.05	0.67±0.04	0.66±0.07	0.56±0.02	0.55±0.01
	Prec.	0.26±0.02	0.62±0.05	0.39±0.03	0.15±0.07	0.45±0.04	0.02±0.00	0.14±0.03	0.34±0.02	0.21±0.02	0.15±0.03	0.62±0.01	0.69±0.02	0.26±0.02	0.15±0.01	0.09±0.02	0.71±0.01	0.62±0.01
	Recall	0.48±0.05	0.66±0.03	0.65±0.04	0.40±0.09	0.60±0.05	0.65±0.09	0.54±0.02	0.62±0.04	0.41±0.06	0.48±0.11	0.53±0.04	0.50±0.05	0.55±0.04	0.43±0.05	0.41±0.04	0.53±0.05	0.50±0.04
	F1	0.33±0.01	0.64±0.02	0.48±0.02	0.22±0.08	0.51±0.03	0.05±0.01	0.22±0.03	0.44±0.01	0.28±0.01	0.22±0.04	0.57±0.02	0.58±0.04	0.35±0.02	0.22±0.01	0.15±0.02	0.61±0.03	0.55±0.02
	AUC	0.64±0.01	0.75±0.04	0.71±0.01	0.61±0.06	0.72±0.03	0.79±0.03	0.64±0.04	0.71±0.01	0.56±0.01	0.61±0.06	0.59±0.00	0.59±0.01	0.65±0.01	0.59±0.02	0.58±0.01	0.60±0.01	0.57±0.01
**CORONET**	*Acc.*	0.72±0.04	0.68±0.06	0.71±0.03	0.74±0.12	0.73±0.06	0.79±0.14	0.73±0.05	0.72±0.03	0.69±0.05	0.68±0.08	0.55±0.04	0.58±0.08	0.72±0.04	0.72±0.05	0.74±0.05	0.57±0.08	0.56±0.05
	Prec.	0.30±0.04	0.60±0.06	0.44±0.05	0.24±0.08	0.51±0.12	0.04±0.02	0.17±0.04	0.39±0.05	0.26±0.04	0.19±0.10	0.61±0.03	0.71±0.05	0.31±0.06	0.18±0.03	0.13±0.02	0.71±0.03	0.63±0.05
	Recall	0.48±0.15	0.66±0.21	0.62±0.17	0.53±0.25	0.59±0.25	0.60±0.12	0.53±0.16	0.61±0.18	0.42±0.14	0.55±0.19	0.49±0.14	0.53±0.17	0.55±0.15	0.44±0.16	0.47±0.14	0.55±0.17	0.46±0.14
	F1	0.37±0.06	0.62±0.14	0.50±0.08	0.31±0.12	0.54±0.18	0.07±0.04	0.26±0.06	0.47±0.09	0.31±0.06	0.27±0.05	0.53±0.10	0.59±0.13	0.39±0.07	0.25±0.05	0.20±0.04	0.61±0.12	0.52±0.11
	AUC	0.70±0.04	0.76±0.07	0.76±0.05	0.69±0.08	0.77±0.09	0.76±0.05	0.70±0.05	0.76±0.06	0.64±0.04	0.70±0.10	0.60±0.03	0.64±0.06	0.72±0.06	0.66±0.05	0.66±0.05	0.62±0.04	0.60±0.05
**SSL-AM**	*Acc.*	0.72±0.03	0.77±0.03	0.74±0.03	0.69±0.06	0.78±0.04	0.75±0.07	0.75±0.02	0.74±0.02	0.68±0.03	0.61±0.07	0.60±0.01	0.63±0.02	0.72±0.03	0.72±0.03	0.75±0.03	0.63±0.02	0.59±0.01
	Prec.	0.32±0.03	0.70±0.03	0.48±0.04	0.18±0.04	0.59±0.06	0.02±0.01	0.20±0.02	0.43±0.03	0.28±0.03	0.16±0.02	0.64±0.01	0.73±0.02	0.34±0.03	0.21±0.02	0.15±0.02	0.74±0.01	0.65±0.02
	Recall	0.57±0.03	0.81±0.04	0.75±0.03	0.48±0.05	0.80±0.08	0.73±0.09	0.59±0.06	0.72±0.03	0.53±0.02	0.60±0.06	0.60±0.02	0.63±0.03	0.66±0.03	0.55±0.05	0.54±0.05	0.65±0.03	0.57±0.02
	F1	0.41±0.03	0.75±0.03	0.58±0.03	0.26±0.05	0.68±0.06	0.04±0.01	0.30±0.03	0.54±0.03	0.36±0.02	0.26±0.02	0.62±0.01	0.68±0.02	0.44±0.03	0.30±0.02	0.24±0.03	0.69±0.02	0.61±0.02
	AUC	0.73±0.03	0.85±0.03	0.80±0.03	0.64±0.05	0.85±0.04	0.78±0.07	0.74±0.04	0.81±0.03	0.67±0.03	0.69±0.08	0.64±0.01	0.67±0.02	0.77±0.03	0.70±0.03	0.72±0.03	0.66±0.02	0.64±0.02
**SSL-AM (ROI)**	*Acc.*	0.68±0.02	0.73±0.01	0.69±0.01	0.65±0.07	0.72±0.03	0.53±0.06	0.73±0.02	0.73±0.01	0.67±0.03	0.63±0.06	0.60±0.02	0.61±0.04	0.70±.2	0.70±0.02	0.72±0.03	0.60±0.03	0.59±0.05
	Prec.	0.28±0.01	0.65±0.02	0.42±0.01	0.18±0.05	0.52±0.03	0.01±0.00	0.18±0.01	0.41±0.03	0.26±0.01	0.21±0.02	0.64±0.01	0.72±0.01	0.32±0.01	0.18±0.02	0.13±0.01	0.72±0.01	0.66±0.01
	Recall	0.56±0.09	0.77±0.06	0.72±0.08	0.55±0.13	0.78±0.03	0.50±0.14	0.54±0.06	0.71±0.06	0.50±0.10	0.82±0.12	0.60±0.08	0.61±0.08	0.63±0.07	0.49±0.13	0.54±0.10	0.63±0.08	0.56±0.08
	F1	0.38±0.03	0.70±0.02	0.53±0.03	0.26±0.07	0.63±0.02	0.02±0.00	0.27±0.01	0.52±0.02	0.34±0.03	0.33±0.02	0.62±0.04	0.66±0.05	0.42±0.02	0.27±0.04	0.21±0.01	0.67±0.05	0.60±0.05
	AUC	0.70±0.04	0.82±0.03	0.77±0.04	0.62±0.07	0.84±0.01	0.56±0.11	0.71±0.02	0.79±0.03	0.66±0.03	0.73±0.04	0.63±0.02	0.66±0.03	0.75±0.03	0.66±0.04	0.70±0.03	0.63±0.03	0.64±0.03

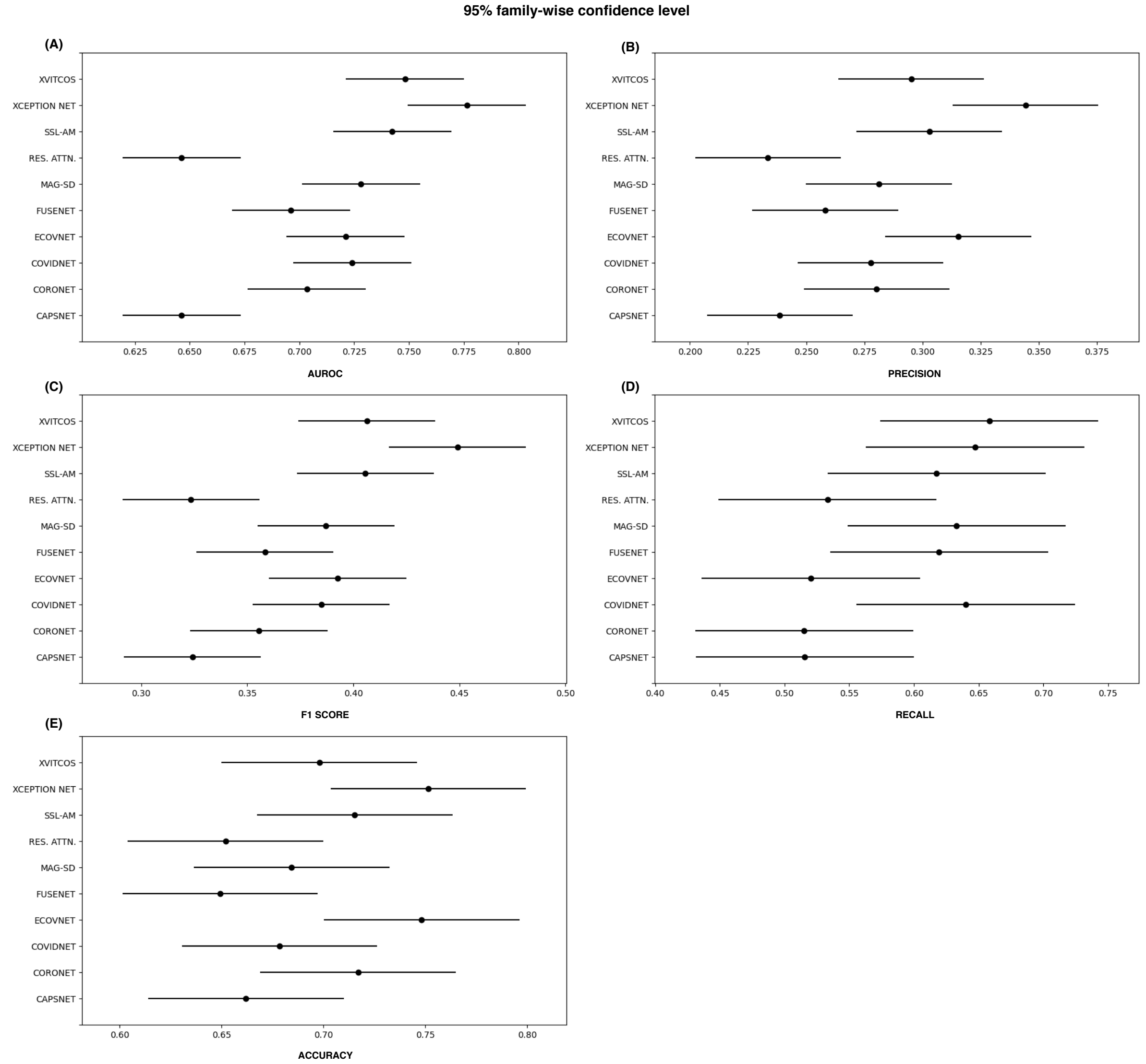

Figure S7: **Tukey test-95% family-wise confidence level for comparison of model performance metrics**. Differences in model performance are evaluated with the following metrics: **(A)** AUROC, **(B)** Precision, **(C)** F1, **(D)** Recall, and **(E)** Accuracy. *Abbrvs: Area Under the Receiver Operator Characteristic (AUROC).*

Table S6: **One-way analysis of variance (ANOVA) for comparison of mean model performance on population subgroups.** *Abbrvs: Accuracy (Acc.); Precision (Prec.); Area Under the Receiver Operator Characteristic (AUROC).*

		**SSL-AM**		**MAG-SD**		**COVIDNET**		**XVITCOS**		**FUSENET**		**CORONET**		**RES. ATTN.**		**ECOVNET**		**CAPSNET**		**XCEPTION NET**	
		F-statistic	*P-value*	*F-statistic*	*P-value*	*F-statistic*	*P-value*	*F-statistic*	*P-value*	*F-statistic*	*P-value*	*F-statistic*	*P-value*	*F-statistic*	*P-value*	*F-statistic*	*P-value*	*F-statistic*	*P-value*	*F-statistic*	*P-value*
**Age**	Acc.	6.4300	0.0000	1.4200	0.2600	2.0600	0.1300	4.9600	0.0100	0.3500	0.8400	1.3100	0.3000	1.6400	0.2000	102.3600	0.0000	6.1800	0.0000	49.0200	0.0000
	AUROC	4.8300	0.0100	11.5200	0.0000	46.8300	0.0000	13.0300	0.0000	28.6700	0.0000	2.3200	0.0900	3.3500	0.0300	17.8500	0.0000	29.0300	0.0000	17.5500	0.0000
	F1	199.8700	0.0000	78.9200	0.0000	183.3100	0.0000	178.3900	0.0000	101.2100	0.0000	21.3900	0.0000	43.8200	0.0000	125.2400	0.0000	121.2800	0.0000	149.3500	0.0000
	Prec.	175.5400	0.0000	102.4400	0.0000	118.8600	0.0000	144.4000	0.0000	68.9700	0.0000	45.2500	0.0000	53.6800	0.0000	223.9200	0.0000	110.5500	0.0000	169.0900	0.0000
	Recall	8.5800	0.0000	4.8000	0.0100	7.3400	0.0000	7.3700	0.0000	2.4500	0.0800	0.9200	0.4700	1.0700	0.4000	9.5900	0.0000	7.3700	0.0000	18.1300	0.0000
**Comorbidity**	Acc.	7.6700	0.0200	3.9500	0.0800	17.7100	0.0000	24.2000	0.0000	6.5300	0.0300	0.2800	0.6100	2.4600	0.1600	2.7000	0.1400	2.2800	0.1700	118.0900	0.0000
	AUROC	6.4400	0.0300	5.5100	0.0500	102.1400	0.0000	25.4400	0.0000	21.1600	0.0000	1.0600	0.3300	9.3300	0.0200	9.6400	0.0100	0.0300	0.8700	132.0600	0.0000
	F1	26.9400	0.0000	6.1400	0.0400	11.6400	0.0100	25.0100	0.0000	3.5200	0.1000	0.4500	0.5200	3.1800	0.1100	10.9300	0.0100	0.1900	0.6700	109.8800	0.0000
	Prec.	73.9700	0.0000	65.4300	0.0000	187.5000	0.0000	87.0800	0.0000	43.3800	0.0000	11.3500	0.0100	59.2800	0.0000	157.6400	0.0000	59.9000	0.0000	276.5400	0.0000
	Recall	4.2400	0.0700	0.5400	0.4800	0.6500	0.4400	0.1200	0.7400	0.3000	0.6000	0.0900	0.7700	0.1700	0.6900	1.7300	0.2300	0.7800	0.4000	8.5900	0.0200
**Ethnicity**	Acc.	3.2700	0.0200	0.7400	0.6000	2.1900	0.0900	2.9100	0.0300	1.3100	0.2900	0.4000	0.8400	5.1300	0.0000	51.2000	0.0000	0.2600	0.9300	52.3700	0.0000
	AUROC	19.1600	0.0000	9.5900	0.0000	24.1500	0.0000	22.5600	0.0000	96.8300	0.0000	1.1200	0.3800	3.8500	0.0100	28.2900	0.0000	8.9300	0.0000	104.8200	0.0000
	F1	74.1200	0.0000	33.6400	0.0000	117.8800	0.0000	107.6800	0.0000	142.6400	0.0000	3.6800	0.0100	24.1000	0.0000	41.7800	0.0000	55.4400	0.0000	56.0500	0.0000
	Prec.	81.7900	0.0000	27.1300	0.0000	61.0000	0.0000	81.0100	0.0000	34.4500	0.0000	13.3000	0.0000	82.8800	0.0000	8.6400	0.0000	55.8700	0.0000	51.8300	0.0000
	Recall	31.0900	0.0000	28.7100	0.0000	13.2500	0.0000	10.9200	0.0000	4.7500	0.0000	0.3900	0.8500	1.4100	0.2600	52.7400	0.0000	13.0000	0.0000	42.8200	0.0000
**Gender**	Acc.	0.2300	0.6400	8.9000	0.0200	10.2800	0.0100	1.6800	0.2300	3.8900	0.0800	0.1600	0.7000	0.3000	0.6000	9.0400	0.0200	0.6600	0.4400	34.9800	0.0000
	AUROC	3.1400	0.1100	3.7300	0.0900	23.2500	0.0000	23.1000	0.0000	1.8100	0.2200	0.2500	0.6300	0.2500	0.6300	7.0800	0.0300	13.4500	0.0100	26.7800	0.0000
	F1	1.0200	0.3400	18.2600	0.0000	18.2700	0.0000	3.0000	0.1200	5.6800	0.0400	1.0400	0.3400	0.9700	0.3500	38.4300	0.0000	6.6800	0.0300	46.8600	0.0000
	Prec.	4.1000	0.0800	44.6300	0.0000	106.6500	0.0000	16.0900	0.0000	30.0400	0.0000	6.8500	0.0300	30.8700	0.0000	176.7200	0.0000	137.2700	0.0000	12.6300	0.0100
	Recall	0.6700	0.4400	7.7300	0.0200	5.7400	0.0400	2.2700	0.1700	1.7800	0.2200	0.6700	0.4400	0.0100	0.9400	23.9600	0.0000	0.6000	0.4600	34.4600	0.0000
**Smoker**	Acc.	1.4600	0.2700	0.3000	0.7400	0.2700	0.7700	0.2800	0.7600	0.0300	0.9700	0.3000	0.7400	0.5200	0.6100	32.1700	0.0000	0.1800	0.8400	4.0400	0.0500
	AUROC	6.9800	0.0100	12.1800	0.0000	31.4000	0.0000	19.8900	0.0000	20.6300	0.0000	2.0500	0.1700	8.1700	0.0100	15.9800	0.0000	23.7800	0.0000	42.3900	0.0000
	F1	56.9300	0.0000	111.7300	0.0000	212.9700	0.0000	189.8600	0.0000	99.3200	0.0000	12.7900	0.0000	104.2500	0.0000	64.5700	0.0000	144.0400	0.0000	244.0800	0.0000
	Prec.	57.9300	0.0000	85.2600	0.0000	87.5600	0.0000	115.0600	0.0000	36.4900	0.0000	24.7700	0.0000	138.2100	0.0000	91.7100	0.0000	79.4300	0.0000	279.6400	0.0000
	Recall	10.3100	0.0000	7.3400	0.0100	2.4400	0.1300	1.4100	0.2800	0.9500	0.4100	0.5300	0.6000	3.9500	0.0500	16.7600	0.0000	12.8400	0.0000	10.0200	0.0000
